# Plant peptidoglycan precursor biosynthesis: Conservation between moss chloroplasts and Gram-negative bacteria

**DOI:** 10.1093/plphys/kiac176

**Published:** 2022-04-26

**Authors:** Amanda J Dowson, Adrian J Lloyd, Andrew C Cuming, David I Roper, Lorenzo Frigerio, Christopher G Dowson

**Affiliations:** School of Life Sciences, University of Warwick, Coventry CV4 7AL, UK; School of Life Sciences, University of Warwick, Coventry CV4 7AL, UK; Centre for Plant Sciences, University of Leeds, Leeds LS2 9JT, UK; School of Life Sciences, University of Warwick, Coventry CV4 7AL, UK; School of Life Sciences, University of Warwick, Coventry CV4 7AL, UK; School of Life Sciences, University of Warwick, Coventry CV4 7AL, UK

## Abstract

Accumulating evidence suggests that peptidoglycan, consistent with a bacterial cell wall, is synthesized around the chloroplasts of many photosynthetic eukaryotes, from glaucophyte algae to early-diverging land plants including pteridophyte ferns, but the biosynthetic pathway has not been demonstrated. Here, we employed mass spectrometry and enzymology in a two-fold approach to characterize the synthesis of peptidoglycan in chloroplasts of the moss *Physcomitrium* (*Physcomitrella*) *patens*. To drive the accumulation of peptidoglycan pathway intermediates, *P. patens* was cultured with the antibiotics fosfomycin, D-cycloserine, and carbenicillin, which inhibit key peptidoglycan pathway proteins in bacteria. Mass spectrometry of the trichloroacetic acid-extracted moss metabolome revealed elevated levels of five of the predicted intermediates from uridine diphosphate *N*-acetylglucosamine (UDP-Glc*N*Ac) through the uridine diphosphate *N*-acetylmuramic acid (UDP-Mur*N*Ac)-D,L-diaminopimelate (DAP)-pentapeptide. Most Gram-negative bacteria, including cyanobacteria, incorporate *meso*-diaminopimelic acid (D,L-DAP) into the third residue of the stem peptide of peptidoglycan, as opposed to L-lysine, typical of most Gram-positive bacteria. To establish the specificity of D,L-DAP incorporation into the *P. patens* precursors, we analyzed the recombinant protein UDP-*N*-acetylmuramoyl-L-alanyl-D-glutamate–2,6-diaminopimelate ligase (MurE) from both *P. patens* and the cyanobacterium *Anabaena sp.* (*Nostoc sp.* strain PCC 7120). Both ligases incorporated D,L-DAP in almost complete preference to L-Lys, consistent with the mass spectrophotometric data, with catalytic efficiencies similar to previously documented Gram-negative bacterial MurE ligases. We discuss how these data accord with the conservation of active site residues common to DL-DAP-incorporating bacterial MurE ligases and of the probability of a horizontal gene transfer event within the plant peptidoglycan pathway.

## Teaser

A functional peptidoglycan biosynthetic pathway in early-diverging land plants is derived from a Gram-negative bacterial progenitor of chloroplasts.

## Introduction

The endosymbiotic theory for the origin of photosynthetic eukaryotes proposes that an engulfed cyanobacterium evolved into the first ancestors of chloroplasts (Dagan et al., 2013; Ponce-Toledo et al., 2017). As with bacteria, these organelles (cyanelles) were surrounded by a peptidoglycan (or murein) wall (Scott et al., 1984). In bacteria, peptidoglycan covers the organism in a mesh-like “sacculus” conferring tolerance to osmotic stress, and a species-specific shape and size. Although originally considered likely that peptidoglycan was lost from all photosynthetic organelles immediately after the glaucophyte branch (Pfanzagl et al., 1996), there has been an accumulation of evidence including sensitivity of chloroplast division to peptidoglycan-directed antibiotics, fluorescent labeling studies, and gene knockout phenotypes to indicate that many streptophytes, including the charophyte algae (Matsumoto et al., 2012; Takano et al., 2018) and some bryophytes (members of the earliest diverging land plant lineage) and pteridophytes (sister lineage to seed plants; Takano and Takechi, 2010; Hirano et al., 2016), may have chloroplasts that synthesize peptidoglycan. Furthermore, in gymnosperms (Lin et al., 2017) and also a diverse number of eudicots (van Baren et al., 2016) all the critical genes for peptidoglycan synthesis have been identified, although a potential penicillin-binding protein (PBP) typically required for peptidoglycan crosslinking has not been confirmed in eudicots.

The earliest evidence for peptidoglycan in embryophytes was uncovered when antibiotics affecting bacterial peptidoglycan synthesis were, in the bryophyte *Physcomitrium patens* (*P. patens*; Kasten and Reski, 1997; Katayama et al., 2003) and lycophytes and ferns (Izumi et al., 2008), found to cause a decrease in chloroplast number with the formation of giant (macro)chloroplasts. Subsequently, genomics and in silico analyses confirmed the presence of all essential bacterial genes for peptidoglycan biosynthesis (Rensing et al., 2008). These genes are nuclear-encoded, predominantly plastid-targeted (Machida et al., 2006; Homi et al., 2009) and transcribed, as revealed by expressed sequence tags. More recently, a peptidoglycan layer surrounding *P. patens* chloroplasts has been visualized using a fluorescently labeled substrate (Hirano et al., 2016) and electron microscopy (Sato et al., 2017).

**Figure 1:**
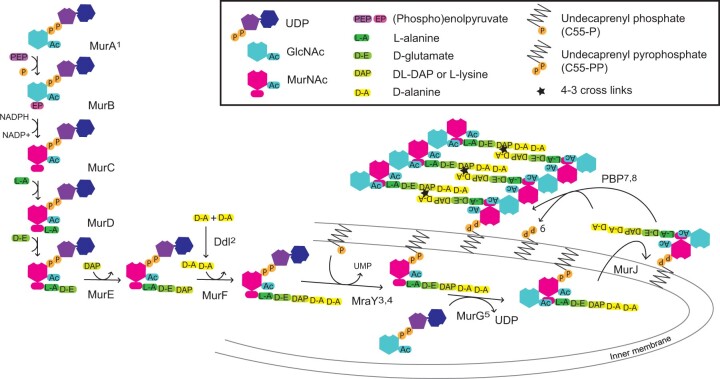
Schematic of the fundamental cytoplasmic and periplasmic enzyme steps in peptidoglycan (murein) biosynthesis. Enzymes: MurA-J, murein synthases A-J; Ddl, D-Ala–D-Ala ligase; MraY, phospho-*N*-acetylmuramoyl-pentapeptide-transferase and PBP, transglycosylase and transpeptidase activities of PBPs. Superscript numbers indicate targets for the following antibiotics: 1, fosfomycin, 2, D-cycloserine, 3, pacidamycin, 4, tunicamycin, 5, murgocil, 6, bacitracin, 7, penicillins, and 8, vancomycin. The cytoplasmic Mur proteins MurA and MurB catalyze the formation of UDP-*N*-acetylmuramic acid (UDP-Mur*N*Ac), Mur ligases (MurC, D, E, and F) sequentially append amino acids to form UDP-Mur*N*Ac-pentapeptide, with commonly either D,L-diaminopimelic acid (D,L-DAP) or L-lysine being incorporated by MurE. The transmembrane protein MraY attaches Mur*N*Ac-pentapeptide to C55-P to yield C55-PP-Mur*N*Ac-pentapeptide (lipid I) and MurG Glc*N*Ac transferase creates C55-PP-Mur*N*Ac-(pentapeptide)-Glc*N*Ac (lipid II). Finally, the disaccharide pentapeptide monomer is flipped into the periplasm, polymerized by the transglycosylase activities of PBPs, or functionally related shape, elongation, division and sporulation (SEDS) proteins, and the peptides are 4-3 cross-linked to pre-existing peptidoglycan by the transpeptidase activities of PBPs or 3-3 cross-linked by L,D-transpeptidases. C55-PP is then subject to pyrophosphatase activity and C55-P recycled.

Peptidoglycan in Gram-negative bacteria has a repeating disaccharide backbone of β-(1,4) linked *N*-acetylglucosamine (Glc*N*Ac) and *N*-acetylmuramic acid (Mur*N*Ac) to which is appended a stem peptide comprising L-Ala, D-Glu, D,L-DAP, D-Ala–D-Ala. Variations in the amino acid residues have been identified and are consequent on either the specificity of the murein ligase enzymes (MurC-F) or later modifications in peptidoglycan biosynthesis. In Gram-positive bacteria, UDP-*N*-acetylmuramoyl-L-alanyl-d-glutamate–2,6-diaminopimelate ligase (MurE) typically incorporates L-Lys as opposed to D,L-DAP, although Bacilli are a notable exception and several other amino acids have been identified in this position (Schleifer and Kandler, 1972; Barreteau et al., 2008; Vollmer et al., 2008). The stem peptides of adjacent saccharide strands are crosslinked by transpeptidation to stabilize the mature peptidoglycan (see biosynthetic pathway Figure 1).

Knockout of *P. patens* homologs of bacterial peptidoglycan synthesis genes D-alanine–D-alanine ligase (*Ddl*), UDP-*N*-acetylglucosamine 1-carboxyvinyltransferase (*MurA*), *MurE*, phospho-*N*-acetylmuramoyl-pentapeptide-transferase (*MraY*), lipid II flippase (*MurJ*), or penicillin-binding protein 1A (*PBP1A*) results in a macrochloroplast phenotype, similar in appearance to antibiotic treatments that target their gene products, while complementation with the intact genes restores the wild-type number of about 50 typical chloroplasts per cell (Machida et al., 2006; Homi et al., 2009; Hirano et al., 2016; Takahashi et al., 2016; Utsunomiya et al., 2020). Cross-species complementation using a *P. patens MurE* (*PpMurE*) knockout showed that the cyanobacterial *MurE* coding sequence from *Anabaena sp.* (*Nostoc sp*. strain PCC 7120; *AnMurE*) fused to the plastid-targeting signal of *PpMurE* can also restore the wild-type chloroplast phenotype (Garcia et al., 2008). In contrast, the homologous Arabidopsis (*Arabidopsis thaliana*) gene, *AtMurE*, failed to complement the *PpMurE* mutant (Garcia et al., 2008). Interestingly, *MurE* knockouts in both *A. thaliana* and maize (*Zea mays*) appear bleached, as opposed to having a macrochloroplast phenotype, are deficient in chloroplast thylakoids and lack many plastid RNA polymerase-regulated chloroplast transcripts, indicating that angiosperm MurE has a primary function in plastid gene expression and biogenesis rather than plastid division (Garcia et al., 2008; Williams-Carrier et al., 2014).

Although data suggestive of the formation of chloroplast peptidoglycan are available, no direct observation of the peptidoglycan precursors or the operation of the chloroplast peptidoglycan synthetic pathway has yet been made. Therefore, here, using pathway-inhibiting antibiotics to drive the accumulation of peptidoglycan intermediates, we establish that in a land plant, *P. patens*, the six Mur proteins and DDL actively synthesize all the main precursors of the peptide stem of peptidoglycan. Furthermore, we show that the pentapeptide building blocks are identical to those of most typical Gram-negative bacteria, including the cyanobacteria, plus the Chlamydiae, the “acid fast” *Mycobacterium* spp. and some Gram-positive bacilli, where D,L-DAP is incorporated instead of L-Lys. Consistent with and supportive of this observation, we show that in vitro the moss MurE ligase, PpMurE, incorporates D,L-DAP in strict preference to L-Lys as the third amino acid within the stem peptide, as would be consistent with the cyanobacterial ancestral origin of the chloroplast, and the enzyme kinetics of PpMurE are similar to cyanobacterial and other Gram-negative D,L-DAP-incorporating MurE ligases.

## Results

### Identification of antibiotics with the most profound effect on *Physcomitrium patens* chloroplast division

**Figure 2:**
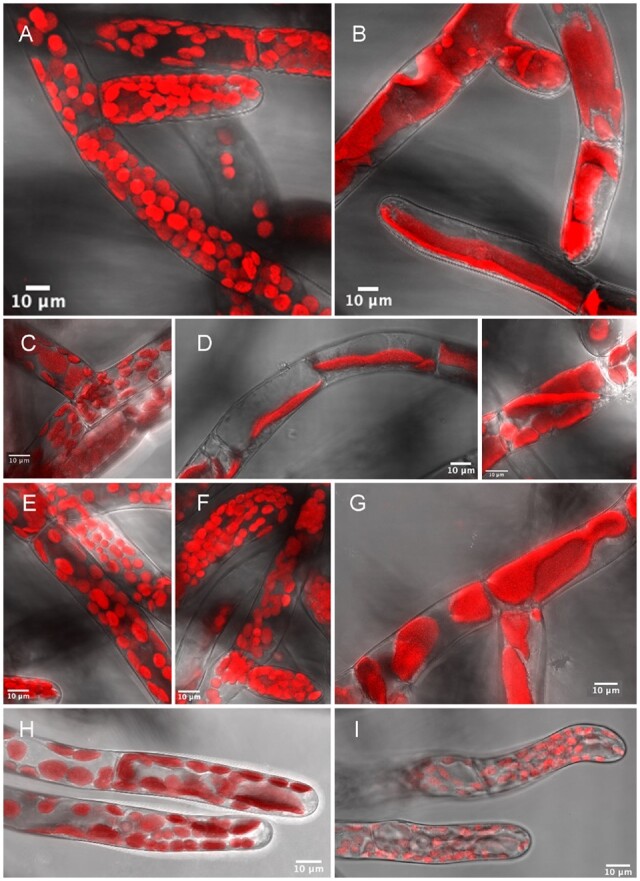
Confocal microscope images showing the effects of antibiotics on *P. patens* chloronemata. Chlorophyll autofluorescence (red) reveals macrochloroplasts consequent on growth on phosphomycin, D-cycloserine, vancomycin, bacitracin, ampicillin, and A22. A, Untreated, B, fosfomycin (500 µg·mL^−1^), C, vancomycin (25 µg·mL^−1^), D, D-cycloserine (20 µg·mL^−1^, two images), E, bacitracin (100 µg·mL^−1^), F, murgocil (10 µg·mL^−1^), G, ampicillin (100 µg·mL^−1^), H, A22 (2.5 µg·mL^−1^), and I, A22 (10 µg·mL^−1^). Sequential fluorescence and transmitted light images, from a Leica SP5 with 63 × oil immersion lens, were processed using LAS AF lite to optimize intensity and combined as hyperstacks using Fiji on Image J. Scale bar: 10 µm.

*Physcomitrium* *patens* was grown on a variety of antibiotics that impact peptidoglycan biosynthesis in bacteria in order to select those best able to cause an accumulation of peptidoglycan intermediates in the moss, so that they might be more readily detected (Figure 1). Of the antibiotics tested the three that appeared most specific at inhibiting peptidoglycan synthesis, as measured by a widespread macrochloroplast phenotype with least effect on chlorophyll intensity, were fosfomycin (500 µg·mL^−1^), a phosphoenolpyruvate analog that inhibits MurA by alkylating an active site cysteine residue, the β-lactam ampicillin (100 µg·mL^−1^), which binds covalently to the active site serine of PBPs, and D-cycloserine (20 µg·mL^−1^), with at least two enzyme targets in peptidoglycan biosynthesis, DDL, and alanine racemase (Figure 2). Even at higher concentrations, where growth rate was impaired, the protonemata were green indicating chlorophyll synthesis and therefore chloroplast function was not substantially impaired. The impact of antibiotics that had either a more profound and pleiotropic effect or that had little impact on phenotype are described in Supplemental Text S1 and include vancomycin, bacitracin, murgocil, and A22 (Figure 2).

### The TCA-extracted metabolome contains peptidoglycan precursors in *Physcomitrium patens*

**Figure 3:**
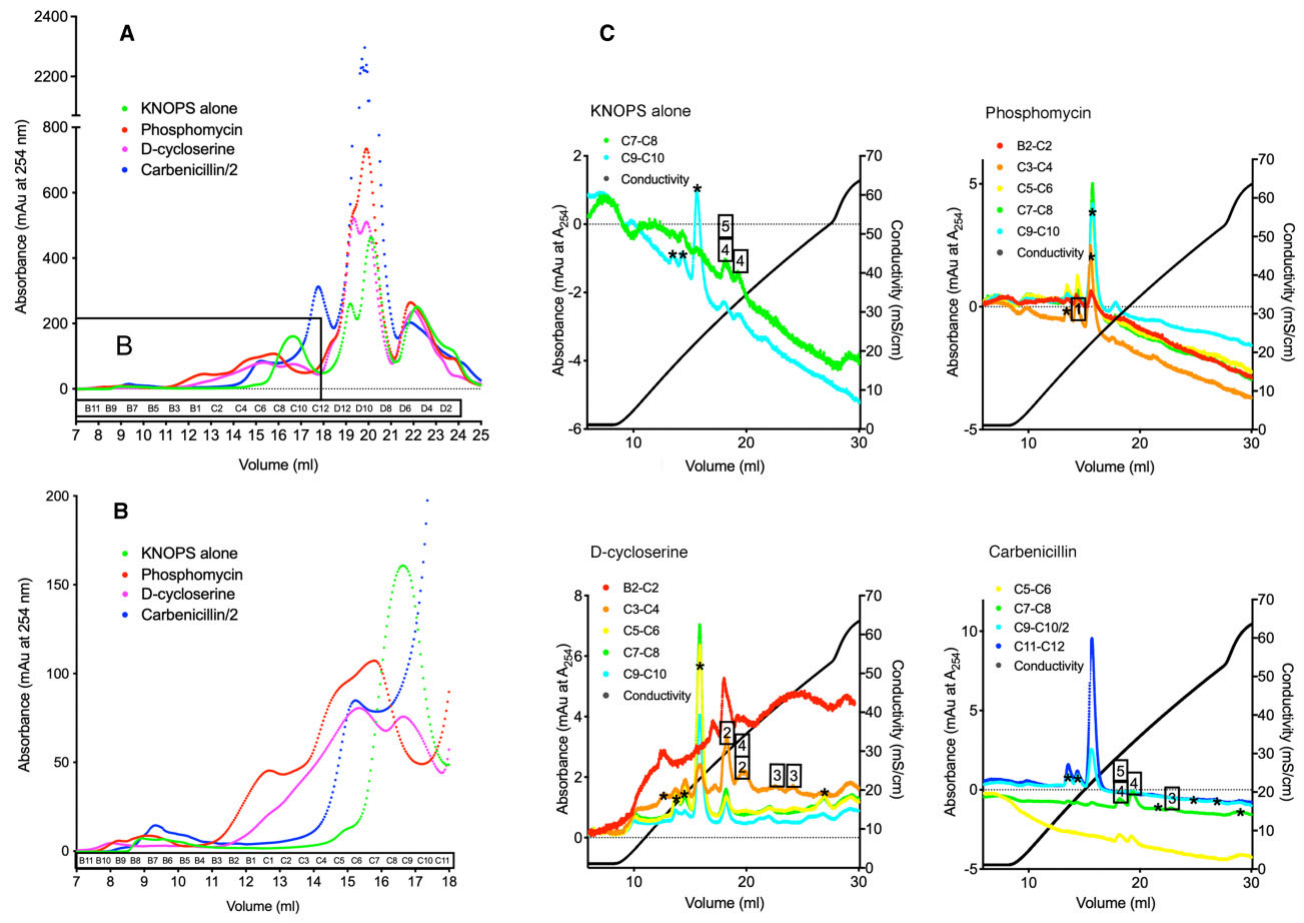
Size exclusion and ion exchange chromatography elution profiles of the TCA-extracted metabolome from *Physcomitrium patens* grown on KNOPS with and without antibiotics. A, Superdex Peptide absorbance traces at 254 nm for the four treatments; KNOPS alone, or KNOPS with fosfomycin (400 μg·mL^−1^), D-cycloserine (100 μg·mL^−1^), or carbenicillin (100 μg·mL^−1^) (for carbenicillin the A254 was divided by two). B, Enlargement of the earlier fractions, where the intermediates were anticipated to elute (as determined by controls). C, MonoQ absorbance traces at 254 nm of pooled Superdex Peptide fractions of 2–20 nmol of UDP species (from B2 to C12). Boxed numbers represent fractions positively identified as intermediates (see Table 1) and asterisks indicate peaks with no recognized component.

*Physcomitrium* *patens* was grown separately on the three most specific and effective antibiotics, fosfomycin (400 µg·mL^−1^), D-cycloserine (100 µg·mL^−1^), and carbenicillin (100 µg·mL^−1^) to facilitate the accumulation of different peptidoglycan precursor molecules (Figure 1). After size exclusion and anion exchange chromatography to purify UDP-linked intermediates from the trichloroacetic acid (TCA)-extracted metabolome, mass spectrophotometric analysis (Supplemental Figure S1) identified precursors common to most Gram-negative bacterial cell wall syntheses (Table 1 and Figure 3, identified precursors numbered 1–5). Precursor molecules were detected only in the earlier fractions from the Superdex Peptide column (Figure 3), as expected from the elution profiles of UDP-Glc*N*Ac and UDPMur*N*Ac-pentapeptide standards.

**Table 1:** UDP-linked intermediates in peptidoglycan biosynthesis as detected by mass spectrometry of the *P. patens* TCA-extracted metabolome, with expected mass:charge (mZ) ratios and actual TOF nanospray values as listed

Peptidoglycan intermediate	Number on MonoQ Trace	Conductivity (mScm^−1^)	Growth medium	Superdex peptide and MonoQ fractions	Species of intermediate detected	Expected mZ	Nanospray TOF value consistent with expected
UDP-Glc*N*Ac	1	18.3	KNOPS + Fos_400_	C3-C4 F15	(m−1)/1	606.0738	606.0814
					(m+Na+−1)/1	628.0557	628.0628
					(m−2)/2	302.5330	302.5352
UDP-Mur*N*Ac-L-Ala	2	29.41 33.77	KNOPS + D-cyclo_100_	C3-C4 F19 *C3-C4 F20*	(m−2)/2	374.0621	374.0696
							*374.0698*
					(m−1)/1	749.1320	749.1476
							*749.1488*
					(m+Na+−1)/1	771.1139	771.1294
							*771.1281*
					(m + 2Na+−1)/1	793.0959	793.1107
							*793.1127*
					(m + 3Na+−1)/1	815.0778	815.0909
							*815.0963*
					(m+Na+−2)/2	385.0531	385.0607
							*385.0611*
UDP-Mur*N*Ac-L-Ala-D-γ Glu	3	44.78 50.97	KNOPS + D-cyclo_100_	C3-C4 F23 *C3-4 F24-25*	(m−2)/2	438.5833	438.5928
							*438.5935*
					(m+Na+−2)/2	449.5744	449.5839
							*449.5858*
					(m + 2Na+−2)/2	460.5653	460.5750
							*460.5716*
	3	41.73	KNOPS + Cb_100_	C7-C8 F23	(m−2)/2	438.583	438.5916
					(m+Na+−2)/2	449.5744	449.5829
					(m + 2Na+−2)/2	460.5653	460.5743
					(m−3)/3	292.0530	292.0575
UDP-Mur*N*Ac*-*L-Ala-D-γ Glu-D,L-DAP	4	29.6 32.74	KNOPS alone	C7-C8 F18-19 *C7-C8 F20*	(m−2)/2	524.6258	524.6289
							*524.6289*
					(m+Na+−2)/2	535.6168	–
							*535.6196*
					(m + 2Na+−2)/2	546.6077	–
							*546.6108*
					(m−3)/3	349.4146	–
							*349.4155*
	4	33.77	KNOPS + D-cyclo_100_	C3-C4 F20	(m−2)/2	524.6258	524.6377
					(m+Na+-2)/2	535.6168	535.6290
					(m + 2Na+−2)/2	546.6077	546.6197
					(m + 3Na+−2)/2	557.5987	557.6108
	4	29.68 32.83	KNOPS + Cb_100_	C7-C8 F19 *C7-C8 F20*	(m−2)/2	524.6258	524.6317
							*524.6324*
					(m+Na+−2)/2	535.6168	535.6230
							*535.6234*
					(m + 2Na+−2)/2	546.6077	546.6139
							*546.6144*
					(m + 3Na+−2)/2	557.5987	–
							*557.6004*
					(m−3)/3	349.4146	349.4173
							*349.4178*
UDP-Mur*N*Ac*-*L-Ala-D-γ Glu-D,L*-*DAP-D-Ala--D-Ala	5	29.6	KNOPS alone	C7-C8 F18-19	(m−2)/2	595.6629	595.6646
					(m+Na+−2)/2	606.6539	606.6553
					(m−3)/3	396.7726	396.7740
	5	29.68	KNOPS + Cb_100_	C7-C8 F19	(m−2)/2	595.6629	595.6693
					(m+Na+−2)/2	606.6539	606.6602
					(m + 2Na+−2)/2	617.6446	617.6509
					(m−3)/3	396.7726	396.7763

Numbers in italics represent species detected in more than one fraction. The enantiomeric forms presented here are those typically identified in Gram-negative bacteria since optical activity cannot be determined by mass spectrometric analysis. *Physcomitrium patens* was grown on KNOPS medium with or without antibiotics, including Fos_400_ (fosfomycin 400 μg·mL^−1^), D-cyclo_100_ (D-cycloserine 100 μg·mL^−1^), and Cb_100_ (carbenicillin 100 μg·mL^−1^). Superdex Peptide (C) and MonoQ fractions (F) where the different species were identified are listed with their peak conductivities on MonoQ, as detailed in in Figure 3. The negative ion nanospray TOF mass spectra from which the data are derived are in Supplemental Figure S1.

The identification of UDP-Mur*N*Ac-Ala-Glu-D,L-DAP in three of the samples as well as the D,L-DAP pentapeptide (Table 1 and Figure 3, numbers 4 and 5), together with the inability to identify UDP-Mur*N*Ac-Ala-Glu-Lys or UDP-Mur*N*Ac-Lys-pentapeptide suggested that in vivo, PpMurE specifically incorporated DL-DAP in the stem peptide third position. By comparison, when the plant was grown on fosfomycin, anticipated to block synthesis of UDP-Mur*N*Ac (Figure 1), only the UDP-Glc*N*Ac precursor was identified (Table 1 and Figure 3, number 1). Interestingly, this metabolite was not detected in the samples treated with the other antibiotics. Similarly, the UDP-*N*-acetylmuramoyl–L-alanine ligase (MurC) and UDP-*N*-acetylmuramoylalanine–D-glutamate ligase (MurD) products, UDP-Mur*N*Ac-Ala and UDP-Mur*N*Ac-Ala-Glu, were detected in the D-cycloserine-grown extract consistent with the accumulation of precursors up to the UDP-*N*-acetylmuramoyl-tripeptide–D-alanyl-D-alanine ligase (MurF) substrate, UDP-Mur*N*Ac-tripeptide (Figure 3, numbers 2, 3, and 4). From the MonoQ anion exchange chromatograms (Figure 3) and the mass spectral data (Supplemental Figure S1), we can conclude that use of the different antibiotics proved to be an effective way to ensure most of the intermediates were detected, confirming the utility of this method for the purpose.

### *Physcomitrium patens* MurE incorporates DL-DAP into the peptidoglycan stem peptide

**Figure 4:**
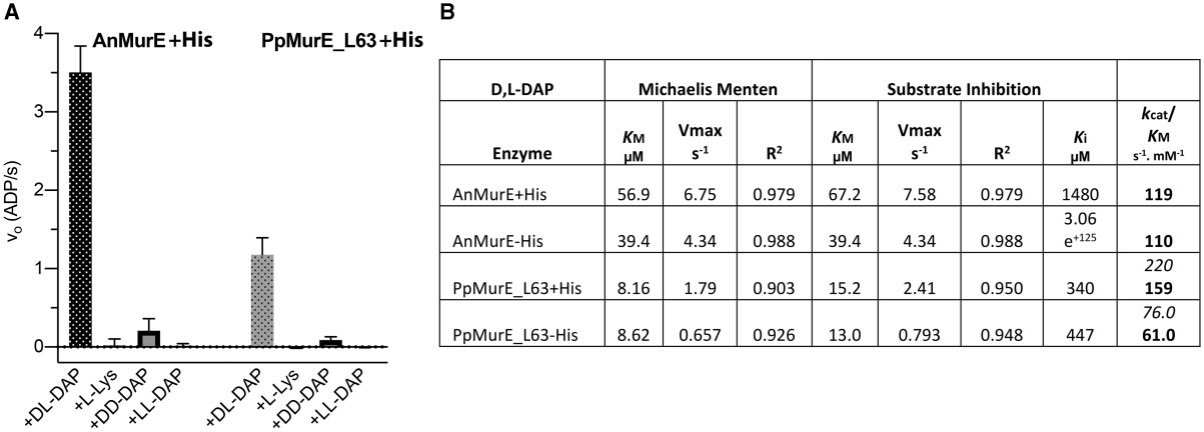
Substrate specificity and D,L-DAP kinetics of AnMurE and PpMurE_L63. A, Activity of AnMurE and PpMurE_L63 with 150-µM D,L-diaminopimelic acid (D,L-DAP), L-Lys, D,D-DAP, or L,L-DAP. Assays included 375-µM UDP-Mur*N*Ac-dipeptide and 100- or 300-nM AnMurE+His or PpMurE_L63+His in 50-mM Hepes pH 7.6, respectively. Results (v_0_) are presented as ADP·s ^−1^ (mols ADP·mol Mur ligase^−1^·s^−1^). Error bars are 95% confidence intervals of five or more rates from up to eight replicate experiments. B, Michaelis-Menten and substrate inhibition values for D,L-DAP: *K*_M_ (μM), V_max_ (ADP·s^−1^), and R^2^ (coefficient for data fit to either model), as computed by Prism, for both enzymes with and without His tags. All constants are “apparent”, obtained at fixed concentrations of the other two substrates. *K*_cat_ derives from *V*_max_ in mols ADP·mol Mur ligase^−1^·s^−1^. *K*_cat_/*K*_M_ values are for Michaelis–Menten kinetics for AnMurE and for substrate inhibition (bold) and Michaelis–Menten kinetics (italics) for PpMurE_L63. (D,L-DAP substrate curves are in Supplemental Figure S2).

To account for the composition of the *P. patens* peptidoglycan stem peptide, we analyzed the activity and substrate specificity of the MurE ligase product of the *PpMurE* gene, with the predicted 62 residue chloroplast transit peptide sequence deleted (PpMurE_L63). The enzyme was compared with the cyanobacterial *Nostoc* MurE ligase. Analysis of the ability of both AnMurE and PpMurE_L63 to utilize D,L-DAP, D,D-DAP, L,L-DAP, and L-Lys revealed that both MurE enzymes were catalytically active in the aminoacylation of UDP-Mur*N*Ac-dipeptide. Removal of the His tag by tobacco etch virus (TEV) protease cleavage did not enhance the efficiency of either enzyme (Figure 4; Supplemental Figure S2) and both proteins highly significantly favored D,L-DAP as a substrate over the other DAP diastereoisomers (unpaired Student’s two-tailed *t* test, *P* ≤ 0.01; Figure 4). Noticeable was the slow but significant rate of turnover of D,D-DAP by AnMurE (*P* ≤ 0.01) and PpMurE_L63 (*P* ≤ 0.05), possibly indicative of a weak stereo-selectivity for the L- over the D stereocenter of DAP utilized by the enzyme when at high concentrations. Importantly, neither enzyme incorporated L-Lys. As a control, lysylation of UDP-Mur*N*Ac-Ala-Glu was also assayed with the L-Lys-specific *Streptococcus pneumoniae* Pn16 MurE (Blewett et al., 2004) and resulted in a rate (vo) of 1.94 ADP·s^−1^ at 150 µM L-Lys, with the same UDP-Mur*N*Ac-dipeptide and ATP concentrations as the other assays.

That the assay followed the aminoacylation of UDP-Mur*N*Ac-dipeptide by D,L-DAP to yield D,L-DAP tripeptide was confirmed by the ability of the assay product to be utilized as a substrate by *Pseudomonas aeruginosa* MurF (PaMurF). This was achieved in the same coupled assay by adding PaMurF at *t* = 0, initiating the MurE ligase reaction with D,L-DAP and then the MurF ligase with D-Ala–D-Ala as the second substrate once the MurE reaction had reached completion to yield the UDP-Mur*N*Ac-pentapeptide (Supplemental Figure S3).

### pH and temperature optima of *Physcomitrium patens* and *Nostoc* MurE

Prior to kinetic investigation of the properties of PpMurE the pH optimum was determined, with that of the cyanobacterial AnMurE, by comparing the rate of ADP generated (v_0_) at pH 5.7–9.7 at approximately saturating concentrations of the substrates (Supplemental Figure S4). Neither of the coupled enzymes in the MurE/ADP release assay was a major factor affecting rate over the pH range studied as evidenced by the independence of the measured MurE rate from coupling enzyme concentration. Additionally, the similarity of activities of the MurE proteins in different buffers allowed us to discount the impact of buffers over the pH range under consideration (Supplemental Figure S4). Assuming saturation with substrates and the only variable responsible for a change in enzyme activity was pH range, we fitted v_0_ versus pH to an equation that follows the relationship of activity to pH. From these data, it was concluded that the optimum for AnMurE is 7.5 and that for PpMurE_L63 is approximately pH 7.5–8.5. The data fit for PpMurE_L63 (*R*^2^ = 0.94 and 0.89, for 1 and 2× coupling enzymes, respectively) is better than that for AnMurE (*R*^2^ = 0.78) indicating that the assumption that other variables (kinetic constants and substrate ionization) are not influenced by pH may be less true for AnMurE.

### *Physcomitrium patens* MurE has similar kinetic properties to cyanobacterial MurE

The two enzymes AnMurE and PpMurE_L63 were assayed to calculate their kinetic efficiency for the preferred substrate, D,L-DAP. From the tabulated data PpMurE_L63 was more sensitive to substrate inhibition from D,L-DAP than AnMurE, as indicated by the greater *R*^2^ value for fit of the PpMurE_L63 data to the kinetics of substrate inhibition compared to the *R*^2^ value for fit to standard Michaelis–Menten kinetics (Figure 4 and the two fitted curves in Supplemental Figure S2). However, the *K*${\text{cat}}_{\;}^{\text{App}}$/*K*_M_^App^ ratio for the plant enzyme were similar to the cyanobacterial one, the most marked difference being the lower D,L-DAP *K*_M_^App^ value, indicative that the plant enzyme may operate at lower substrate concentrations in vivo. These figures were compared with reported data for other MurE activities (Supplemental Table S1) and reveal that the plant and cyanobacterial MurE are at least as catalytically active, as indicated by the *K*${\text{cat}}_{\;}^{\text{App}}$/*K*_M_^App^ ratio, as the bacterial homologs.

### Conservation of amino acid residues common to DL-DAP-incorporating MurE ligases

BlastP searches and sequence alignments indicated that the bacterial MurE homolog sharing closest sequence identity with PpMurE_L63 is that of the *Gemmatimonadetes bacterium* RMH74196.1 (50.0 percentage identity), a photoheterotrophic Gram-negative bacterium in a phylum quite distal to the cyanobacteria (Zeng et al., 2014). The next closest was the MurE of the Gram-positive *Bacillus sp. HF117* (44.2%), which would be anticipated to incorporate D,L-DAP (Barreteau et al., 2008). Both share considerably more sequence identity with PpMurE than the cyanobacterial AnMurE (37.8%), determined in this article to be D,L-DAP incorporating, *Escherichiacoli* MurE^D,L-DAP^ (34.9%) and *Mycobacterium tuberculosis* MurE^D,L-DAP^ (34.7%). The L-Lys incorporating enzymes, all from Gram-positive species, share still less similarity: *Thermatoga maritima* MurE^L-Lys^ (33.0%), *Streptococcus pneumoniae* MurE (30.1%) and *Staphylococcus aureus* MurE^L-Lys^ (26.6%). Likewise, an evolutionary phylogram computed by the Maximum Likelihood method (Supplemental Figure S5) placed AnMurE and MurE of other cyanobacteria as more distantly related than *Gemmatimonadetes* to plant MurE, as represented by PpMurE_L63 and the algal streptophytes. The alga *Mesotaenium endlicherianum* (66.2%) represents a late charophyte ancestor within the Zygnematophyceae, which are predicted to be on a branch point preceding embryophyte evolution (Donoghue and Paps, 2020), whereas *Klebsormidium nitens* (56.1%) and *Coleochaete scutata* (62.3%) in the Klebsormidiophyceae and Coleochaetaceae, respectively, and also within the charophyte algae, are on more divergent branches.

To relate homology to functionality, PpMurE was aligned in Clustal Omega (EMBL-EBI) with homologs of both L-Lys- and DL-DAP-incorporating MurE ligases (Supplemental Figure S6). Many amino acid residues are conserved not only between MurE from bacteria and streptophytes but also across the Mur ligase family of proteins (as indicated by asterisks on Supplemental Figure S6). Mur ligases comprise three domains: an N-terminal Rossmann-fold domain responsible for binding the UDP-Mur*N*Ac substrate, a central ATP-binding domain, and a C-terminal domain associated with binding the incoming amino acid. Most of the amino acids conserved between the different Mur ligases lie within the central ATP-binding domain, those in the N- and C-termini commonly do not co-localize across the Mur ligase family with the known substrate binding residues of a particular ligase.

Amino acids of published importance for ATP binding (species abbreviation subscripted); the P-loop within TGTXGKT^Sa^, E220^Mt^, D356^Sa^, N347^Mt^, R377^Mt^, and R392^Mt^ are conserved in the plant enzymes *M. endlicherianum* MurE and PpMurE, as well as a lysine, K219^Sa^, carbamylated in MurD for positioning the MgATP complex for the generation of a transient UDP-Mur*N*Ac-phosphodi-peptide intermediate (Dementin et al., 2001). K360^Sa^ and Y343^Mt^ have undergone conservative changes. Similarly, residues that bind UDP-Mur*N*Ac, S28^Ec^, HQA45^Ec^, NTT158^Ec^, E198^Mt^, S184^Ec^, QXR192^Ec^, and H248^Mt^ are no less conserved in these streptophyte MurE homologs than they are between bacteria.

Although most of the UDP-Mur*N*Ac-tripeptide interactions are within the MurE central domain, those made in relation to the appended amino acid, D,L-DAP or L-Lys, are within the C-terminal domain. All of the identified bacterial MurE residues that interact with D,L-DAP are highly conserved in the *M. endlicherianum* and *P. patens* proteins. More specifically, with reference to *E. coli* MurE and *M. tuberculosis* MurE, it is possible to distinguish those that interact with either the D- or L-stereocenter carboxylates of D,L-DAP: G464^Ec^, E468^Ec^, D413^Ec^, and N414^Ec^, which bond to the D-stereocenter, R389^Ec^, which bonds with the L-stereocenter, and especially R416^Ec^, which interacts with both the L- and D-center carboxylates. Of these R389^Ec^, N414^Ec^, R416^Ec^, G464^Ec^, and E468^Ec^ are less consistently present in MurE ligases from Gram-positive bacteria that incorporate L-Lys, a decarboxylated derivative of D,L-DAP, which has only been reported to interact with the R383^Sa^, D406^Sa^, and E460^Sa^ residues (Ruane et al., 2013). Similarly, the pattern of charged residues in the C-terminal domain of these streptophyte MurE homologs (those highlighted red or purple in Supplemental Figure S6) would indicate a binding cleft for the amino acid substrate that is more basic and resembles that of the Gram-negative MurE ligases. Together these data are in complete accord with our kinetic findings that D,L-DAP is the preferred substrate in moss and cyanobacterial MurE, rather than L-Lys. As would be anticipated from the phylogeny, the more closely related *G. bacterium* MurE aligns strongly with the Gram-negative DL-DAP incorporating enzymes, and includes the DNPR motif, which confers specificity for the D-stereocenter carboxyl and amino groups of D,L-DAP, indicating that this phylum is most likely to incorporate DL-DAP.

## Discussion

### *Physcomitrium patens* peptidoglycan is synthesized from a UDP-Mur*N*Ac-D,L-DAP-pentapeptide

Growth of *P. patens* on the antibiotics fosfomycin, D-cycloserine, and ampicillin facilitated the detection, by mass spectrophotometric analysis of the TCA-extracted metabolome, of peptidoglycan intermediates up to UDP-Mur*N*Ac-Ala-Glu-DAP-Ala-Ala in the moss. These data enable us to conclude that the identical basic building blocks for the Gram-negative bacterial cell wall are found in non-vascular embryophytes. With evidence for knockout phenotypes for *P. patens* homologs of bacterial *MraY*, *MurJ*, and *PBP1A* and the presence of mRNA for UDP-*N*-acetylglucosamine–*N*-acetylmuramyl-(pentapeptide) pyrophosphoryl-undecaprenol *N*-acetylglucosamine transferase (*MurG*; Machida et al., 2006; Homi et al., 2009; Utsunomiya et al., 2020) it would be expected that the D,L-DAP-containing pentapeptide within the stroma is lipid-linked then flipped across the chloroplast inner envelope membrane and polymerized into peptidoglycan to form a “sacculus” bounding the organelle, as indicated from fluorescent-labeling using a D-Ala–D-Ala analog (Hirano et al., 2016). By analogy with bacteria and from the predicted transit peptides of the peptidoglycan-maturing proteins it is anticipated that the peptidoglycan will lie between the inner and outer membranes of the chloroplast, although this has yet to be determined (Figure 1).

### PpMurE appends D,L-DAP to UDP-Mur*N*Ac-Ala-Glu

From our data, it is evident that the moss MurE ligase, with the transit peptide omitted, PpMurE_L63, can efficiently append D,L-DAP to UDP-Mur*N*Ac-L-Ala-D-Glu in vitro, as can the cyanobacterial enzyme from *Nostoc* sp. strain PCC 7120, AnMurE. This latter is in accordance with the D,L-DAP content of peptidoglycan in the cyanobacteria *Synechococcus* sp. and *Synechocystis* sp. (Jurgens et al., 1983; Woitzik, 1988) and is inconsistent with the observation that *Anabaena cylindrica* may incorporate L-Lys (Hoiczyk and Hansel, 2000). Our in vitro MurE enzymological data also complement the mass spectrometric analysis of the antibiotic-grown *P. patens*, which identified UDP-Mur*N*Ac-D,L-DAP intermediates as being present in vivo in the TCA-extracted metabolome.

That UDP-Mur*N*Ac-L-Ala-D-Glu is an efficient substrate for PpMurE_L63 is important in that there is no obvious homolog in most green plants for glutamate racemase (MurI), exceptions include the glaucophyte alga *Cyanophora paradoxa* (Contig25539), the charophyte alga *K. nitens* (GAQ85716.1) but not *M. endlicherianum*, a zygnematophycean alga in the extant Zygnematophyceae lineage that is sister to the embryophyte lineage. Here, this function may be replaced by a D-alanine amino transferase (DAAA), of which there are two genes having weak similarity to *Bacillus subtilis* DAAA in both *P. patens* and *M. endlicherianum* (Phytozome v.13 *P. patens*: Pp3c6_5420 (15.7%), Pp3c16_17790 (14.7%) and OneKP *M. endlicherianum*: WDCW scaffolds 2009723 (17.6%) and 2007189 (16.5%)). Alternatively *P. patens* diaminopimelate epimerase (DapF), like Chlamydial DapF, may possess the dual specificity required to racemize L-Glu to D-Glu in addition to its epimerization of L,L-DAP to D,L-DAP (De Benedetti et al., 2014).

### Substrate preference of AnMurE and PpMurE

The high degree of specificity of both AnMurE and PpMurE_L63 for D,L-DAP, over the alternatives L,L-DAP, D,D-DAP, and L-Lys, is consistent with other D,L-DAP-incorporating enzymes assayed in vitro, including *E. coli* MurE, *M. tuberculosis* MurE, and *Chlamydia trachomatis* MurE, for which L-Lys is either a very poor substrate or is not accepted at all (Supplemental Table S1). Similarly, the L-Lys-incorporating *S. aureus* MurE does not incorporate D,L-DAP in vitro. Not all MurE ligases are as selective, *Thermotoga maritima* MurE incorporates L-Lys and D-Lys in almost equal amounts in vivo (Huber, 1986) and can efficiently incorporate D,L-DAP in vitro (Boniface et al., 2006). In this regard, it is notable that *T. maritima* MurE possesses a DDP**R** motif, which includes the arginine residue of the consensus DNPR of D,L-DAP-incorporating enzymes which hydrogen bonds to and stabilizes D,L-DAP, consequently the almost complete absence of D,L-DAP in *T. maritima* peptidoglycan has been attributed to its low intracellular concentration. This almost absolute specificity of most MurE ligases is indicative of a requirement that the stem peptide be composed of the correct amino acids to facilitate optimal transpeptidation (Vollmer et al., 2008).

### PpMurE is a slow but efficient MurE ligase

Kinetic analyses of PpMurE_L63 demonstrated an enzymatic efficiency similar to bacterial MurE homologs, as estimated by comparison of *K*_cat_^App^/*K*_M_^App^ (Supplemental Table S1). Further comparisons with other D,L-DAP-incorporating enzymes, and in particular those of the obligate intracellular pathogens *C. trachomatis* and *M. tuberculosus*, revealed the plant MurE to have a similarly low *K*_M_ for the amino acid substrate relative to the L-Lys-incorporating enzymes. This may reflect either (or both) a lower abundance of D,L-DAP or the potential toxicity of the D,L-diamino acid, particularly in a eukaryotic cell (Kolukisaoglu and Suarez, 2017). A higher *K*_M_ for L-Lys-incorporating MurE ligases has been attributed to the much greater abundance of this amino acid in bacteria (Mengin-Lecreulx et al., 1982; Ruane et al., 2013).

The availability of the D,L-DAP substrate in plants, as in cyanobacteria and Chlamydiae, is not in question as the biosynthesis of L-Lys is catalyzed by DAP decarboxylase (LysA) from D,L-DAP which is ultimately derived from aspartate (Hudson et al., 2006).

Comparison of the PpMurE_L63 *K*_cat_^App^ with the bacterial enzymes reveals the rate of turnover to be quite low, possibly reflecting the apparent low density of peptidoglycan surrounding the chloroplast and a concomitant slower rate of synthesis compared to rapidly dividing, free-living bacteria. Moreover, the plant enzyme has a dependence on D,L-DAP best fitted to a substrate inhibition model, possibly to ensure that peptidoglycan precursor biosynthesis proceeds at a rate consistent with protein synthesis, which utilizes D,L-DAP in L-Lys formation.

### A second MurE-like protein in *P. patens*

**Figure 5:**
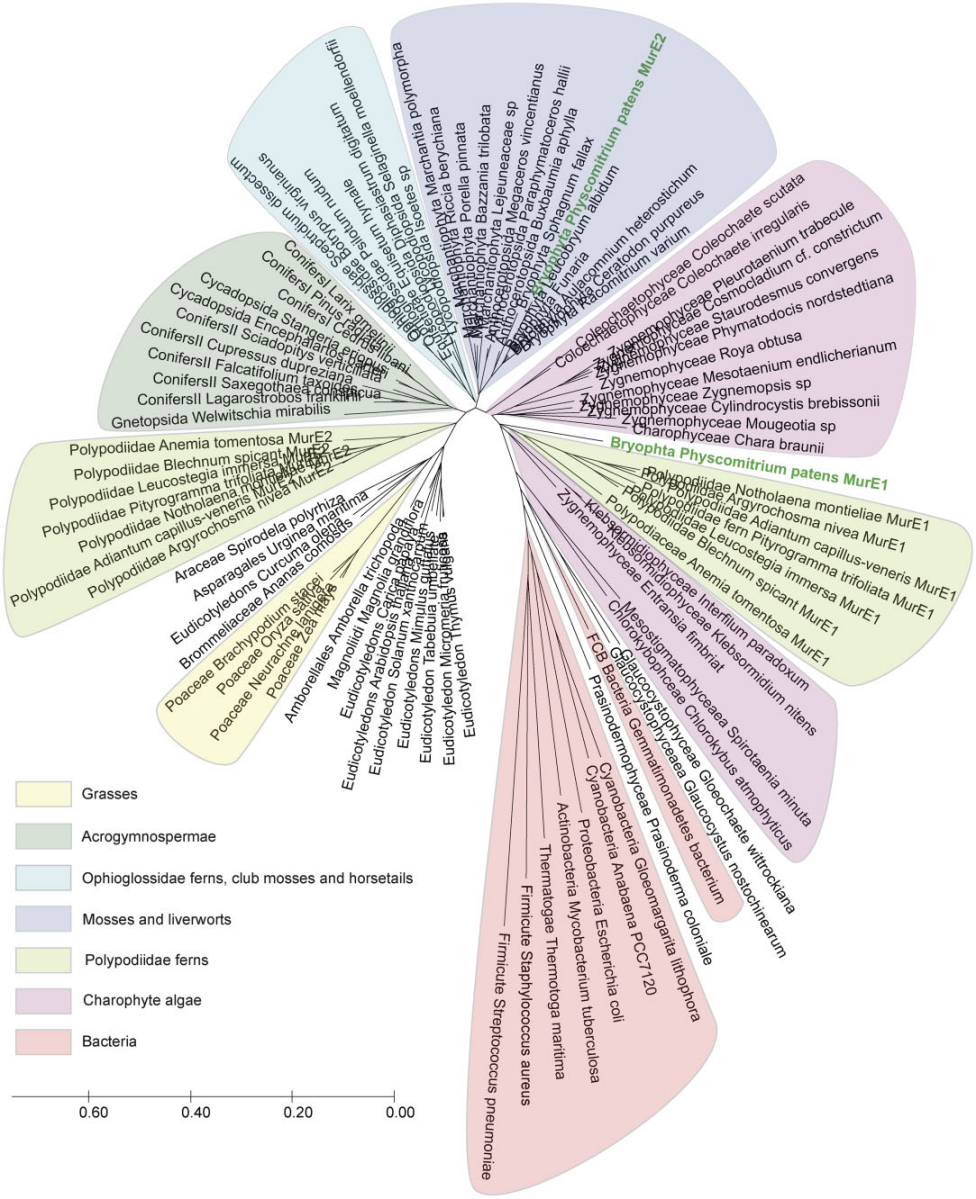
Evolutionary relationships of the two PpMurE proteins to representative MurE homologs. *Physcomitrium patens*, as well as many ferns in the Polypodiidae, encodes two MurE homologs: PpMurE1 and PpMurE2, labeled in bold. Different taxonomic groups are boxed to highlight the relationships of the *P. patens* proteins to bacterial, algal, and embryophyte phyla. The evolutionary history was inferred using the Minimum Evolution method and computed using MegaX software. The evolutionary distances are in units of the number of amino acid substitutions per site. PpMurE1 and the shorter Polypodiidae fern “MurE1” homologs have shorter branch lengths to charophyte algae than other land plants. PpMurE2, which includes a long, quite unstructured, amino terminal domain, and a conserved carboxy terminal extension, shares closest identity with most marchantiophytes and other bryophytes, which lack a second MurE homolog, whereas the longer Polypodiidae “MurE2” proteins map more closely with the Acrogymnsopermae and Magnoliopsida.

It is important to mention that the *P. patens* genome encodes two MurE homologs (PpMurE1: Pp3c23_15810, studied in this article, and PpMurE2: Pp3c24_18820), which have 72.2% amino acid identity to each other over the conventional bacterial MurE ligase domains and 48.4% identity overall. Due to the synteny between *P.patens* chromosomes 23 and 24, it would be anticipated that MurE was duplicated as a consequence of the first whole genome duplication event identified in bryophytes (Lang et al., 2018). RNA sequence data indicate that the expression profiles of the two genes are at variance, although viewed overall they are expressed to similar levels (Phytozome v13 and *P. patens* efp browser; Ortiz-Ramirez et al., 2016),

PpMurE2 primarily differs from PpMurE1 in having a long extension at the amino terminus and a short, carboxy terminal extension. The relationship of the two proteins was investigated by phylogenetic analysis (Figure 5), whereby a similar duplication of MurE was identified in the Polypodiidae ferns but not most bryophytes. The amino terminal extension of PpMurE2 is approximately 290 residues longer than most bacterial MurE homologs, compared to 94 residues for PpMurE1, and is common to the MurE-like homologs identified in most seed plants, as well as MurE of some streptophyte algae and nonseed plants. This region of unknown function, beyond any predicted transit peptide, is typically rich in acidic residues and structure predictions for the apoform indicate it to be unfolded. The carboxy terminal extension is highly conserved across the streptophytes; in PpMurE2, it extends to 24 residues beyond a consensus streptophyte DDREECREAL motif present also in PpMurE1 (Supplemental Figure S6).

Although the DNPR motif and other amino acids associated with D,L-DAP binding are retained in PpMurE2, knockout mutations of PpMurE1 alone results in a comprehensive macrochloroplast phenotype (Machida et al., 2006; Garcia et al., 2008), consistent with the hypothesis that this protein is sufficient for peptidoglycan synthesis in the moss. Moreover, preliminary in vitro experiments indicate that PpMurE2 does not function as a MurE ligase and we would suggest that both the amino and carboxy terminal extensions have been acquired during streptophyte evolution to participate in different protein:protein or protein:nucleic acid interactions thereby facilitating the development of an alternative function for MurE within the chloroplast transcription and translation apparatus.

In contrast to *P. patens* many in the same and closely related phylla, including the Acrogymnospermae, encode a single *MurE* homolog with amino and carboxy terminal extensions similar to PpMurE2 yet these proteins would be anticipated to function as MurE ligases since their genomes encode most of the peptidoglycan synthesis enzymes (Lin et al., 2017). We propose the shorter MurE in *P. patens* and the Polypodiidae ferns represent de-evolution of streptophyte MurE to more closely resemble its bacterial counterpart. It has yet to be determined at what point in streptophyte evolution the function of MurE changed and whether in any plants it remains a bifunctional protein capable of both MurE ligase activity and interaction with chloroplast RNA polymerase in chloroplast transcription (Garcia et al., 2008).

The retention of a DNPR motif is common not only in the nonseed plants but also most seed plant MurE homologs, with the similarly charged DNPK motif also being common, and the Poaceae and a few Pinaceae being notable exceptions (DNPA and DNSR, respectively). That embryophyte MurE has evolved an alternative function essential to plastid photomorphogenesis in seed plants indicates an exaptation from its original function in peptidoglycan biosynthesis and plastid division (Williams-Carrier et al., 2014). This raises the intriguing question why important residues of the D,L-DAP-binding motif are retained, in similar proximity to the ATPase domain, in these proteins.

### Predicted streptophyte peptidoglycan structure from peptidoglycan gene homologies

The moss “sacculus”, like that of Chlamydiae, has been recalcitrant not only to visualization by electron microscopy but also to common extraction protocols, making analysis of the mature polymer a future goal. The moss chloroplast envelope membranes were found to be closely appended with little dense intervening material (Takano and Takechi, 2010; Matsumoto et al., 2012; Sato et al., 2017), likewise in Chlamydiae, the apparent deficit of a bounding sacculus led to the term the “chlamydial anomaly” (Packiam et al., 2015). This is in marked contrast to most cyanobacteria where the cell wall is highly crosslinked and forms a broad, electron dense layer (Hoiczyk and Hansel, 2000). Intermediate between these extremes is the earliest divergent lineage in plant evolution, the glaucophyte algae, where the cyanelles are bounded by a peptidoglycan layer that has been more tractable to visualization and analysis (Pfanzagl et al., 1996; Higuchi et al., 2016).

It would appear that progressive transition of a bacterium from free-living to endosymbiont or pathogen and thence to an integrated organelle is associated with a reduction in substance of the sacculus. Presumably there are not the same osmotic constraints and risks of dehydration within the host cell and the vestigial peptidoglycan may function primarily or exclusively for the purpose of assembly of the division apparatus. Additionally, it may be that for cyanobacterial evolution into a cyanelle and subsequently a plastid that a finer, net-like cell wall would be a prerequisite if extensive exchange of larger molecules, including lipids and proteins, were to occur. Supportive of this suggestion is the fact that most of the bacterial PBPs which crosslink the lipid-linked Glc*N*Ac-Mur*N*Ac-pentapeptide precursor, where identified in the *P. patens* genome, the sequences encode only partial proteins or have no predicted product from RNA-seq data (Leebens-Mack et al., 2019). Currently, the only reported exception is a PBP1A homolog, the transpeptidase and transglycosylase functions of which have an almost complete knockout phenotype in *P. patens* (Machida et al., 2006; Takahashi et al., 2016).

We also propose that streptophyte peptidoglycan must differ in its mature form by being uniquely modified to distinguish it from the peptidoglycan of potential plant pathogens. The *P. patens* genome encodes a battery of proteins that include peptidoglycan-binding and LysM domains (more than 35 of which are identified in Phytozome v13). Many of these proteins will be part of the defenses of the plant cell which are activated on detection of fungal and bacterial cell wall material. To evade the host cell defenses it is anticipated that an endosymbiont, obligate pathogen or evolving organelle must protect its peptidoglycan from the host enzymes, conceivably by modification of the peptide stem (Wolfert et al., 2007) or the Glc*N*Ac-Mur*N*Ac backbone (Davis and Weiser, 2011). Predictions as to what those modifications might be in streptophytes are hampered by the fact that the ancestry of the modifying enzymes is not necessarily cyanobacterial. We have reported here the closer similarity of PpMurE to MurE in the Gemmatimonadetes phylum and we can further include *P. patens* PBP1A, MurF, MurD, MurG, and Ddl as having closest sequence identity to homologs within the same Fibrobacteres–Chlorobi–Bacteroidetes group of Gram-negative bacteria. In accord with these findings, the diverse origins of the majority of *P. patens* peptidoglycan biosynthesis proteins has been reported, with MurA and MraY being the exceptions with closer identity to cyanobacterial homologs (Sato and Takano, 2017). Therefore, it appears highly probable that a horizontal gene transfer event of a distinct Gram-negative peptidoglycan-related gene cluster must have occurred early in the plant lineage. Hence, we conjecture a simultaneous transfer of peptidoglycan-modifying genes could have occurred that would introduce modifications to the mature polymer, distinct from any in cyanobacteria. This is not without precedent, as the divergent glaucophyte algae were found to append *N*-acetyl-putrescine to the second residue in the stem peptide (Pfanzagl et al., 1996).

Here, we have determined that chloroplast peptidoglycan in the streptophyte, *P. patens*, is constructed from typical Gram-negative UDP-Mur*N*Ac-D,L-DAP-pentapeptide peptidoglycan precursor. However, we propose that the final polymerized structure derived from this building block differs from its cyanobacterial progenitor by being both less highly polymerized and, to distinguish it from plant pathogens and thereby evade the plant immune response, substantially modified.

## Materials and methods

### Plant material

*Physcomitrium patens* Gransden strain, GrD13) was grown on modified KNOPS with 5-mM diammonium tartrate, to promote chloronemata formation (Schween et al., 2003). The medium was solidified with 0.85% (w/v) plant agar (Sigma) and overlaid with 9-cm cellophane discs (AA Packaging). Plants were grown in 90-mm diameter × 20-mm vented tissue culture dishes sealed with Micropore (3M) surgical tape in a plant growth room at 21°C under continuous light from Sylvania white F100W tubes at 65–100 μmol·m^−2^·s^−1^. After being homogenized axenically in water in a 250-mL flask using an IKA T18 digital Ultra Turrax homogenizer, for one to two 12 s bursts, *P. patens* protonemata were cultured as 2-mL aliquots per 25-mL solid KNOPS plus tartrate.

### Confocal microscopy of antibiotic-treated *P. patens* protonemata

Confocal single plane images and Z-series stacks were acquired on a Leica SP5 microscope, using a 63 × 1.4 Oil UV immersion objective with the 405-nm laser line, at 19% intensity, offset −0.3% and gain 700 and transmitted light, offset −0.2% and gain 190, with photo multiplier tube spectral detection adjusted for chlorophyll emission (628–800 nm). Images were processed using the Fiji distribution of ImageJ v2.0.0.

### TCA extraction of plant metabolites

Antibiotics were added to KNOPS plus tartrate agar at 100-µg·mL^−1^ carbenicillin, 100-µg·mL^−1^ D-cycloserine, or 400-µg·mL^−1^ fosfomycin. After 15 days, tissue was harvested, weighed, and ground in liquid nitrogen using a pestle and mortar before being frozen at −80°C. To extract TCA-soluble plant metabolites the tissue was ground again in 5 mL·g^−1^ of ice cold 10% (w/v) TCA (Fisons AR grade) before being mixed gently in 50-mL Falcon tubes on a rolling shaker for 30 min at 4°C (Roten et al., 1991). Insoluble material was pelleted at 48,000*g*, 10 min, 2°C, the supernatant was retained and the pellet re-extracted twice more, first with 2.5 mL·g^−1^ and then with 1.25 mL·g^−1^ (of the original pellet weight) of ice cold 10% (w/v) TCA. The pooled supernatants were extracted into an equal volume of diethyl ether, to remove TCA, by manually shaking for 3 × 20 s in a separating funnel before recovering the lower, aqueous layer. The ether extraction of the aqueous phase was repeated twice more. The pH of the combined lower phases was restored to pH 7–8 using 1-M NaOH and residual ether was removed in vacuo at which point, the sample was lyophilized.

### Purification of muropeptide precursors

The nucleotide precursors in the TCA-soluble metabolite extracts were first partially purified by size exclusion using a Superdex Peptide 10/300GL column. The freeze-dried pellets were resuspended in deionized water, applied to the column as a 500-µL aliquot, eluted with deionized water, and collected as 0.5-mL fractions at 0.5 mL min^−1^. The likely elution volume of molecules of interest was established by elution of 20-nmol UDP-Mur*N*Ac-DAP-pentapeptide and 20-nmol UDP-*N*-acetyl-glucosamine (Sigma) standards.

The *A*_260_ of pooled Superdex Peptide fractions of 1–2 mL was used to determine the upper limit of the total concentration of UDP species and an estimated 2-, 10-, or 20-nmol UDP species in 2-mL 10-mM ammonium acetate, pH 7.5, was loaded onto a MonoQ 50/5 GL column equilibrated in the same buffer. Bound molecules were eluted with a 27-mL linear gradient of 10 mM to 0.81-M ammonium acetate (pH 7.5), at 0.7 mL·min^−1^ and collected as 1-mL fractions using an Äkta Pure where the eluate absorbance was recorded at *A*_230_, *A*_254_, and *A*_280_. Peaks with an absorbance ratio of usually 1:2 A_280_:A_254_ were selected for freeze drying and mass spectrometry.

### Mass spectrometry nanospray time-of-flight analysis of peptidoglycan UDP-Mur*N*Ac precursors

Identity of UDP-Mur*N*Ac precursors were confirmed by negative ion time-of-flight (TOF) mass spectrometry using a Waters Synapt G2Si quadrupole-TOF instrument operating in resolution mode, equipped with a nanospray source calibrated with an error of less than 1 ppm with sodium iodide over a 200–2,500 *m/z* range (Catherwood et al., 2020). Samples, freeze dried three times to remove ammonium acetate, were diluted in liquid chromatography mass spectrometry (LCMS) grade 50% v/v acetonitrile to between 1 µM and 5 µM. They were introduced into the instrument using Waters thin wall nanoflow capillaries and up to 20 min of continuum data were collected at a capillary voltage of 2.0 kV, cone and source offset voltages of 100V and 41V, respectively. Source and desolvation temperatures were 80°C and 150°C, respectively, desolvation and purge gas flow rates were both 400 L·min^−1^. Scan time was 1 s with an interscan time of 0.014 s. Scans were combined into centered mass spectra by Waters Mass Lynx software. Resolution (m/z/half-height spectral peak width) was measured as 1 in 20,100.

### Construction of heterologous expression plasmids

*PpMurE* (derived from the full-length Pp3c23_15810V3.2 EST clone) and *AnMurE* (derived from *Anabaena sp.* [*Nostoc* sp. strain PCC 7120 *MurE* WP_010995832.1 Q8YWF0|MURE_NOSS1]) were inserted into the vector pPROEX HTa (Addgene) in order to be expressed in frame with an amino terminal, TEV protease-cleavable, hexa-histidine (His6) tag. The *MurE* coding sequences were polymerase chain reaction (PCR) amplified from their respective DNA (Machida et al., 2006; Garcia et al., 2008) in pTFH22.4 using the primers PpMurE_L63_Forward (TTTGCGACATGTTGAAAATGGGGTTTGGGGATTCGAAATTGACGGATCG) and PpMurE_Reverse (AAACGCGCGGCCGCTTATTTTCTAAGTCGCAAAGCCTCCCGACATTCCTC) and Anabaena_PCC7120_MurE_Forward (TTTGCGGGTCTCTCATGAAATTGCGGGAATTACTAGCGACAGTAGACAGTG) and Anabaena_PCC7120_MurE_Reverse (AAACGCGCGGCCGCTTATAATTTTTCTCTTTCTGTCAAAGCGGCGCGTGCG). The amplified region for *PpMurE* started at leucine 63, effectively deleting the chloroplast transit peptide at the cleavage site predicted by the ChloroP1.1 Prediction server (Emanuelsson, 1999) and introducing an unique *Nco1*-compatible *Pci1* site around the ATG and a *Not1* site immediately 3′ to the stop codon. A TAA stop codon was substituted for the native TGA. The *AnMurE* primers amplified the cDNA and *Bsa1* and *Not1* sites were introduced 5′ and 3′ to the ATG start and TAA stop codons, respectively. The former was sited to create an *Nco1*-compatible 5′-cohesive end. The vector pPROEX HTa was restricted with *Nco1* and *Not1* and gel purified before being ligated to *Pci1-Not1* restricted *PpMurE_L63* or *Bsa1-Not1* restricted *AnMurE* PCR fragments that had been cleaned up with a PCR clean up kit (Qiagen). Coding sequences were confirmed by Sanger sequencing (Eurofins).

### Expression of *PpMurE_L63* and *AnMurE* and protein purification

For protein purification *E.coli* strains were tested for optimal expression: BL21 ([DE3] Thermofisher) was selected for PpMurE_L63_pPROEX and Tuner cells ([DE3] Novagen), with the chaperone plasmid pG-KJE8 (Takara Bio Inc.), were selected for AnMurE_pPROEX. These were grown in L-Broth plus 0.2% v/v glucose, 100 µg·mL^−1^ ampicillin and 35 µg·mL^−1^ chloramphenicol at 37°C to an *A*_600_ of 0.6 when *PpMurE* expression was induced with 0.5-mM IPTG and *AnMurE* expression was induced by 0.5-mM IPTG with 1.5 mg·mL^−1^ arabinose and 8 ng·mL^−1^ tetracycline to induce pG-KJE8 chaperones. Bacteria were then grown overnight at 19°C, harvested by centrifugation at 5,600*g*, 15 min at 4°C and resuspended in Buffer A: 50-mM HEPES–NaOH, 0.5-M NaCl, 10-mM imidazole, and 10% v/v glycerol (pH 7.5) containing EDTA-free protease inhibitor tablets, as recommended by the supplier (Pierce), and 2.5 mg·mL^−1^ lysozyme, with gentle mixing for 30 min at 4°C. Lysis was by sonication on ice for 10 × 15 s bursts at 70%, interspersed by 1–2 min cooling on ice. Insoluble material was pelleted at 50,000*g* for 30 min at 4°C and the supernatant loaded directly onto a 5-mL His Trap HP (GE Healthcare) at 2-mL·min^−1^ and washed with 50-mL Buffer A at 4 mL·min^−1^ at 4°C. Bound material was eluted with a 100-mL linear gradient to 100% Buffer B: 50-mM HEPES–NaOH, 0.5 M NaCl, 5% w/w glycerol, and 0.5-M imidazole (pH 7.5) at 4 mL·min^−1^. Selected peak fractions were pooled and concentrated in either 30- or 50-kDa MWCO Vivaspin concentrators (GE Healthcare), for AnMurE or PpMurE_L63, respectively, at 2,800*g* at 4°C. Proteins were further purified by size exclusion chromatography on Superdex G200 XK26 (GE Life Sciences) pre-equilibrated and eluted with 50-mM HEPES–NaOH, 150-mM NaCl (pH 7.5) and purity of the eluted MurE proteins was established by sodium dodecyl sulfate–polyacrylamide gel electrophoresis (Supplemental Figure S7). Pooled peak fractions were dialyzed against DB2: 30-mM HEPES–NaOH, 1-mM MgCl_2_, 50-mM NaCl, 50% v/v glycerol with 0.2-mM PMSF, 1-µM leupeptin, 1-µM pepstatin, 3-mM dithiothreitol (pH 7.6) overnight at 4°C, before storage at −20°C and −80°C.

### TEV protease-cleaved protein preparation

Bacteria were harvested and lysed using a cell disruptor and the proteins first purified on 5-mL His Trap HP columns, using Buffer A and B (pH 8.0) as above, except that 100-mM Tris replaced 50-mM HEPES and Buffer A included 2% v/v glycerol, 10 mg·L^−1^ DNase1 (DN25), and 1-mM DTT. Pooled fractions were exchanged into a buffer of 50-mM PIPES, 100-mM NH_4_SO_4_, 200-mM KCl, 20-mM MgCl_2_, 1-mM DTT, 30-mM imidazole, 2% v/v glycerol (pH 7.7) using a stack of four 5-mL HiTrap Desalting columns (Pharmacia). Peak fractions were incubated for 48h at 4°C in the ratio 1 mg TEV protease: 50-mg protein before reverse His-tag purification, collecting the column flow through. Samples were concentrated using 50-kDa concentrators as above.

*Streptococcus pneumoniae* MurE and PaMurF were overexpressed and purified exactly as described (Blewett et al., 2004; Majce et al., 2013).

### Mur ligase activity assays

The assays employed a continuous spectrophotometric method following ATP consumption at 37°C in a Cary 100 UV/Vis double beam spectrophotometer. Mur ligase catalyzed ADP release, coupled to NADH oxidation by pyruvate kinase and lactate dehydrogenase, led to stoichiometric consumption of NADH measured by a fall in the *A*_340_. Assay volumes were 0.2 mL and contained 50-mM PIPES, 10-mM MgCl_2_ adjusted to pH 6.7 for AnMurE or 50-mM Tricine, 10-mM MgCl_2_ adjusted to pH 8.7 for PpMurE_L63, 1-mM dithiothreitol, 0.2-mM NADH, 2-mM phosphoenol pyruvate, 1-mM ATP, 50-mM·min^−1^ pyruvate kinase, and 50-mM·min^−1^ lactate dehydrogenase (as assayed by the manufacturer, Sigma). Ligases were diluted prior to assay as required in 50-mM HEPES (pH 7.7), 50-mM KCl, 1-mM MgCl_2_, 3-mM DTT, 50% v/v glycerol, 0.2-mM PMSF. Uridine diphospho *N*-acetylmuramyl-L-alanyl-D-glutamate (UDP-Mur*N*Ac-Ala-Glu) was synthesized by recapitulation of the segment of the peptidoglycan synthesis pathway responsible for the conversion of UDP-Gluc*N*Ac to UDP-Mur*N*Ac-Ala-Glu and purified by anion exchange chromatography (Lloyd et al., 2008). Concentrations of UDP-Mur*N*Ac dipeptide, Mur ligase, and amino acid substrates were as described in the text or table legends. Control rates were collected usually in the absence of the amino acid, or UDP-Mur*N*Ac-dipeptide as specified, and the activity of the enzyme was initiated by addition of the missing component. Mur ligase initial rates were recorded as mol ADP·mol Mur ligase^−1^·s^−1^ (ADP/s) within the linear range of the time course of the assay.

### Phylogenetic analyses

Sequences were sourced from Phytozome v13 (Goodstein et al., 2012), OneKP (Leebens-Mack et al., 2019), NCBI, and Uniprot databases. Percentage identities were computed using Clustal Omega with M View (EMBL-EBI; Brown et al., 1998) and Minimum Evolution phylogenetic analyses (Rzhetsky and Nei, 1992) using ClustalW and MegaX software (Kumar et al., 2018).

### Accession numbers

Sequence data from this article can be found in the GenBank/EMBL data libraries under accession numbers BAE45863.1 and RUR85277.1.

## Supplemental data

The following materials are available in the online version of this article.

**Supplemental Text S1.** Effects of antibiotics on *P. patens.*

**Supplemental Figure S1.** Negative ion nanospray TOF mass spectra of TCA-extracted peptidoglycan intermediates.

**Supplemental Figure S2.** D,L-diaminopimelic acid (D,L-DAP) substrate curves for AnMurE and PpMurE_L63.

**Supplemental Figure S3.** Assay data demonstrating *Pseudomonas aeruginosa* MurF (PaMurF) utilizes the products of AnMurE and PpMurE_L63.

**Supplemental Figure S4.** Activities of AnMurE and PpMurE_L63 with pH and buffer.

**Supplemental Figure S5.** Maximum Likelihood phylogram of MurE ancestry using bacterial and algal species with. *P. patens* mature MurE protein (PpMurE_L63), computed using ClustalW and MEGAX software.

**Supplemental Figure S6.** Clustal Omega (EMBL-EBI) multiple sequence alignment of MurE homologs displayed using Jalview with ClustalX designated colors.

**Supplemental Figure S7.** PAGE gel of AnMurE and PpMurE_L63 after gel filtration.

**Supplemental Table S1.** Comparison of AnMurE and PpMurE_L63 kinetics with D,L- diaminopimelic acid (D,L-DAP) with published data for other MurE ligases.

## Supplementary Material

kiac176_Supplementary_Data

## Notes

A.J.D. generated the heterologous expression vectors, prepared and assayed proteins, purified peptidoglycan intermediates from *P. patens*, and wrote the manuscript; A.J.L. conceived the use of TCA extraction, performed the mass spectrophotometric analysis and analyzed the data; A.C.C. assisted with *P. patens* handling; D.I.R, and L.F initiated the enzymatic analysis and Pump Prime funding, C.G.D. assisted with funding and A.J.L., D.I.R., and C.G.D. assisted with experimental discussion and critiqued the manuscript.

The author responsible for distribution of materials integral to the findings presented in this article in accordance with the policy described in the Instructions for Authors (https://academic.oup.com/plphys/pages/general-instructions) is: A.J. Dowson (a.j.dowson@warwick.ac.uk).
