# Supplementary material for: Plant peptidoglycan precursor biosynthesis: Conservation between moss chloroplasts and Gram-negative bacteria
Source: Plant Physiol. 2022 Apr 26;190(1):165–79. doi: 10.1093/plphys/kiac176 (PMC9434261; doi:10.1093/plphys/kiac176)
Supplement: kiac176_Supplementary_Data [file kiac176_supplementary_data.pdf]

Supplemental Text S1 Effects of antibiotics on *P. patens*

Antibiotics which only rarely effected macrochloroplast development in *P. patens* included vancomycin (1.0, 5.0 and 25 μg.ml^{-1}), a 1.45 kDa glycopeptide which binds D-Ala--D-Ala, thereby inhibiting PBP transpeptidation, and the 1.42 kDa bacitracin (20 and 100 μg.ml^{-1}), which complexes with C55-isoprenyl pyrophosphate, inhibiting recycling (Figure 1[8,6] and Figure 2, C and E). Neither antibiotic typically traverse cytoplasmic membranes, and therefore a strong phenotype was not expected as it is anticipated they would have to penetrate not only the cytoplasmic membrane but also, potentially, the outer chloroplast membrane. It may be that any observed effect of these antibiotics was restricted to damaged or senescing cells. At high concentrations (500 μg.ml^{-1}) bacitracin did cause premature senescence.
A22 hydrochloride, a smaller molecule at 271.6 kDa, was tested at 2.5 and 10 μg.ml^{-1} and was likewise found to result in macrochloroplast formation in some but not most cells, although at higher concentrations its impact was more pleiotropic and chloroplasts were considerably bleached (Figure 2, H and I). A22 inhibits MreB, an actin homolog and cytoskeletal protein that controls bacterial width in rod-shaped bacteria by spatiotemporal regulation of peptidoglycan synthesis. Since there is not an evident MreB homolog in the moss (Ozdemir et al., 2018), any effect of A22 may be consequent on a less specific effect on chloroplast heat shock proteins having homology to MreB, especially HSP70 (Gao and Gao, 2011).
Another antibiotic clearly pleiotropic in its effect was tunicamycin (0.2, 1.0 and 5.0 μg.ml^{-1}), a glycoprotein that inhibits the transfer of phospho-Mur*N*Ac-pentapeptide to the lipid carrier undecaprenyl pyrophosphate by MraY (Figure 1[4]). At concentrations equal to or above 1 μg.ml^{-1} it caused chloroplast malformation, slow growth and apoptosis. This could be attributed to its effect on the maturation of glycoproteins in the endoplasmic reticulum since, in eucaryotes, tunicamycin also blocks the transfer of UDP-Glc*N*Ac to dolichol phosphate.
Pacidamycins 1 and 5, cationic peptides with homology to the bacteriophage øX174 lysis protein Arg-Trp-x-x-Trp motif, believed to bind the cytoplasmic surface of MraY and thereby inhibiting it (Figure 1[3]) (Rodolis et al., 2014; Bugg and Kerr, 2019), had little effect on either growth rate or chloroplast division. Likewise, Murgocil, a 448 Da steroid-like molecule, which inhibits peptidoglycan synthesis in *Staphylococcus aureus* and is predicted to bind in the MurG active site blocking UDP-Glc*N*Ac access (Figure 1[5]), when tested at 1, 5 and 25 μg.ml^{-1} was found to have little effect on protonemata phenotype (Figure 2, F).
The effect of the three antibiotics, fosfomycin, D-cycloserine and ampicillin (Figure 2 B, D and G), subsequently selected for investigating the accumulation of peptidoglycan intermediates, is detailed in the text of the paper.

Bugg TDH, Kerr RV (2019) Mechanism of action of nucleoside antibacterial natural product antibiotics. J Antibiot (Tokyo) 72: 865-876
Gao H, Gao F (2011) Evolution of the chloroplast division machinery. Frontiers in Biology 6: 398-413
Ozdemir B, Asgharzadeh P, Birkhold AI, Mueller SJ, Rohrle O, Reski R (2018) Cytological analysis and structural quantification of FtsZ1-2 and FtsZ2-1 network characteristics in Physcomitrella patens. Sci Rep 8: 11165
Rodolis MT, Mihalyi A, Ducho C, Eitel K, Gust B, Goss RJ, Bugg TD (2014) Mechanism of action of the uridyl peptide antibiotics: an unexpected link to a protein-protein interaction site in translocase MraY. Chem Commun (Camb) 50: 13023-13025

Supplemental Figure S1 Negative ion nanospray TOF mass spectra of TCA-extracted peptidoglycan intermediates, with the expected mass:charge (mz) values for the different species in red boxes. *P. patens* was grown on KNOPS medium with and without antibiotics, including Fos$_{400}$ (Fosfomycin 400 µg.ml^{-1}), D-cycloserine$_{100}$ (D-cycloserine 100 µg.ml^{-1}) and Cb$_{100}$ (carbenicillin 100 µg.ml^{-1}). UDP-linked intermediates were purified by size exclusion chromatography on Superdex Peptide columns (fractions labelled C) followed by ion exchange on MonoQ columns (fractions labelled F). Column elution profiles are in Figure 3 and the compiled data in Table 1.

UDP-Glc*N*Ac

KNOPS + Fos$_{400}$ (C3-4 F15)

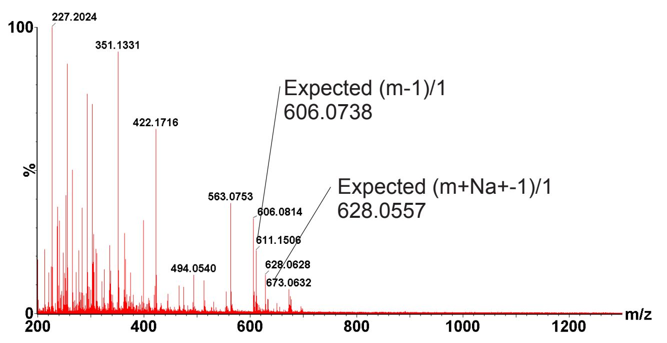

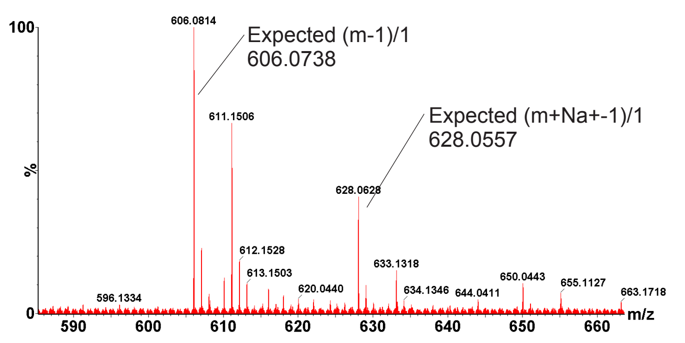

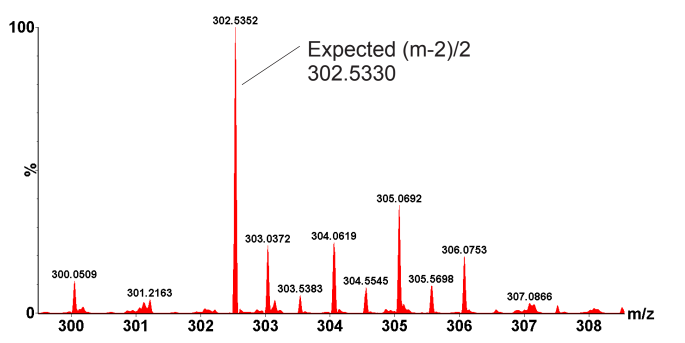

UDP-Mur*N*Ac-Ala

KNOPS + D-cycloserine$_{100}$ (C3-4 F19)

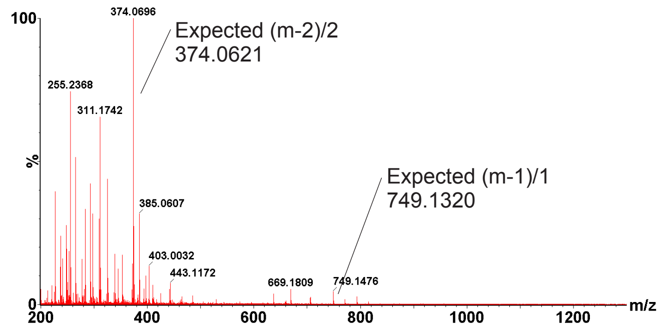

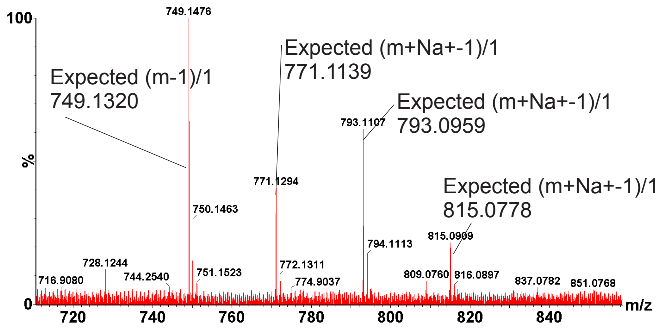

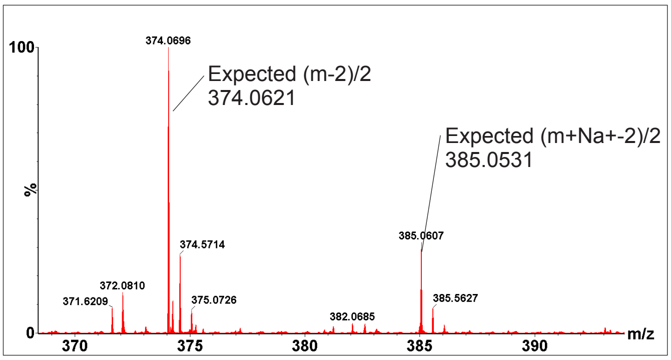

KNOPS + D-cycloserine$_{100}$ (C3-4 F20)

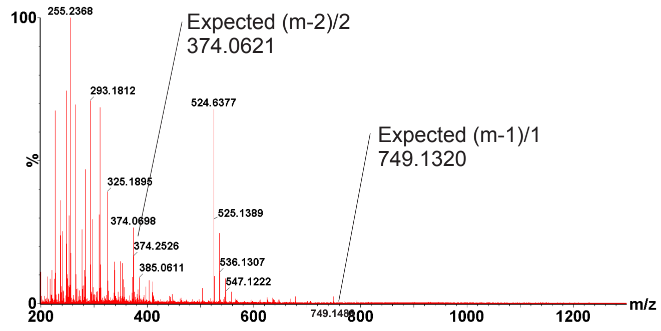

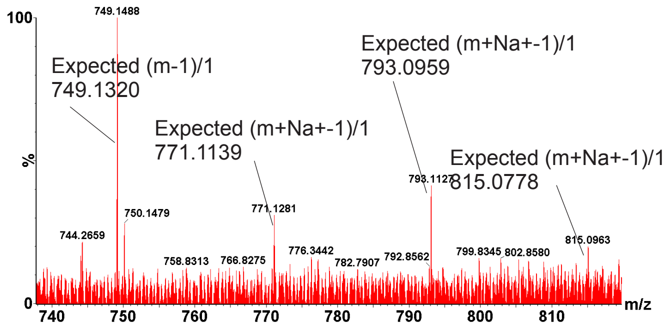

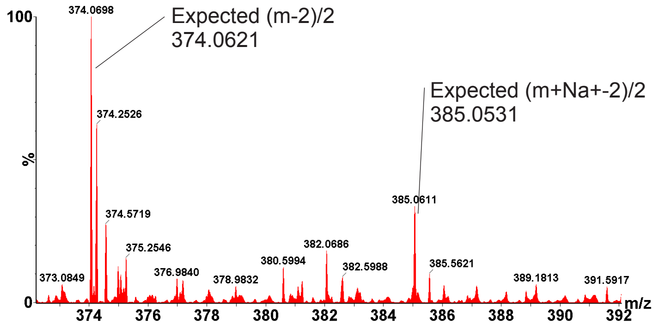

UDP-Mur*N*Ac-dipeptide (Ala:Glu)

KNOPS + D-cycloserine$_{100}$ (C3-4 F23)

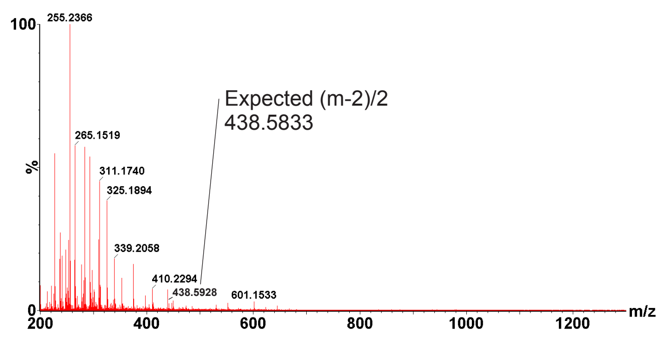

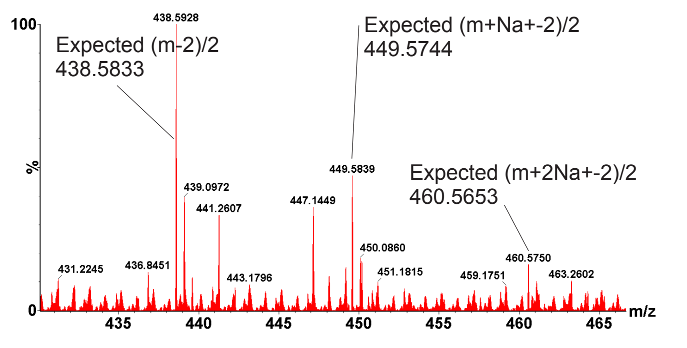

KNOPS + D-cycloserine$_{100}$ (C3-4 F24-25)

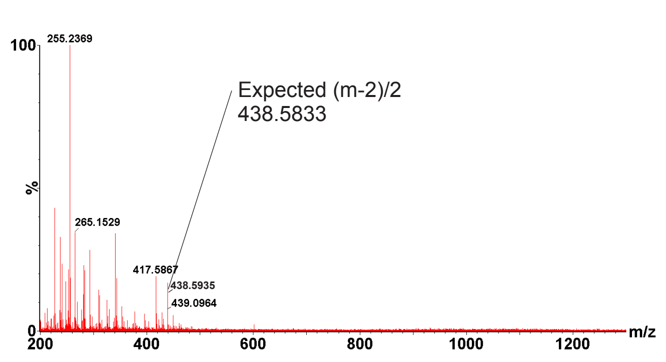

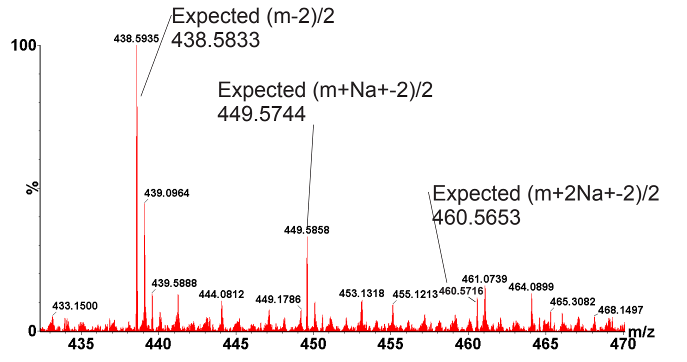

KNOPS +Cb$_{100}$ (C7-8 F23)

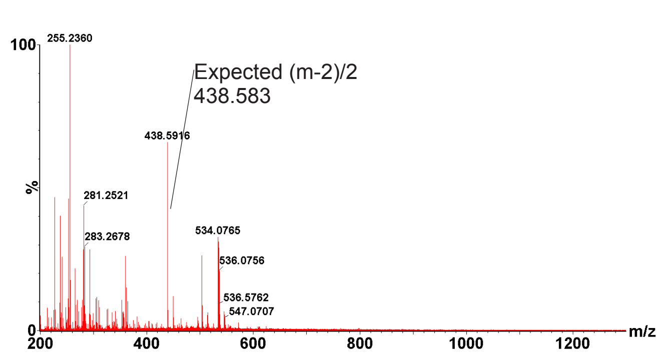

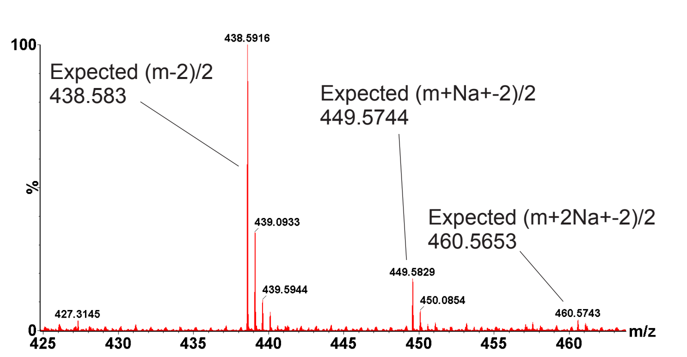

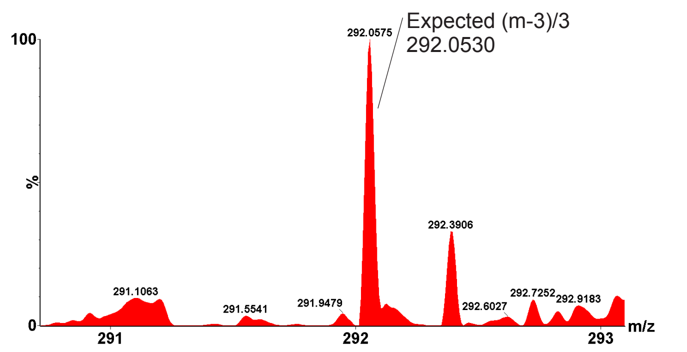

UDP-Mur*N*Ac-tripeptide (Ala:Glu:DAP)

KNOPS alone (C7-C8 F20)

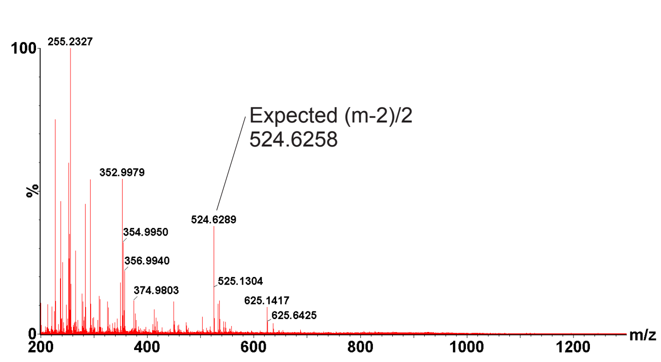

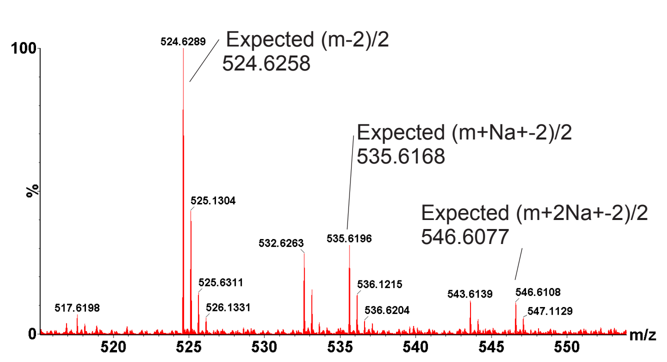

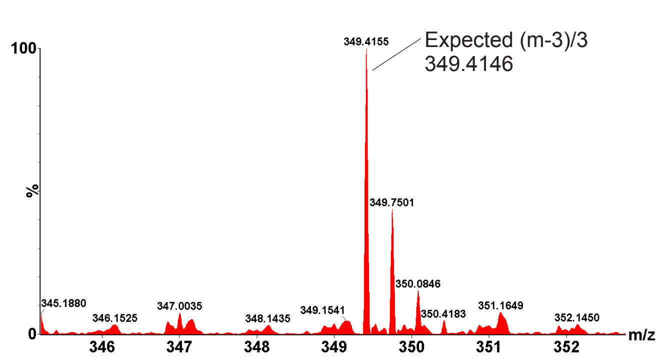

KNOPS + D-cycloserine$_{100}$ (C3-4 F20)

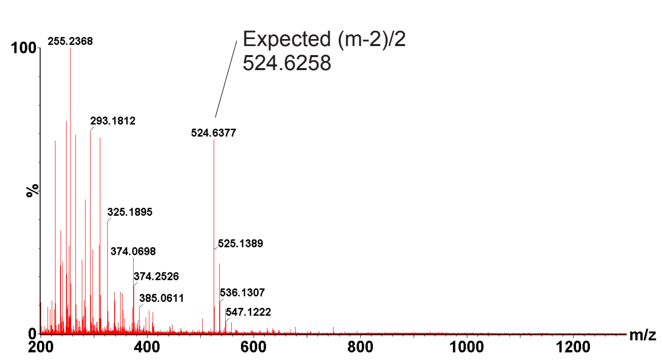

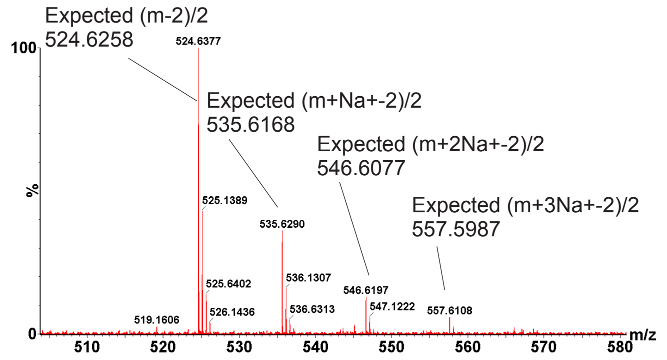

KNOPS +Cb$_{100}$ (C7-8 F19)

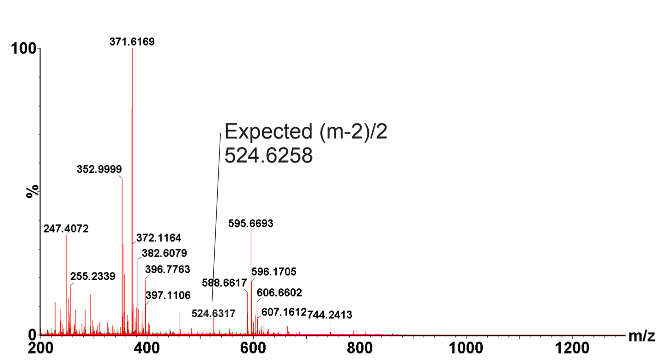

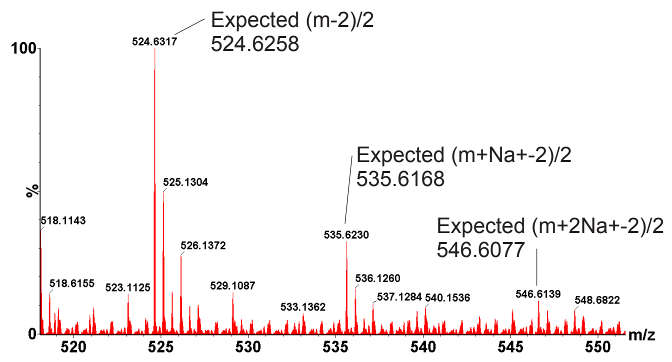

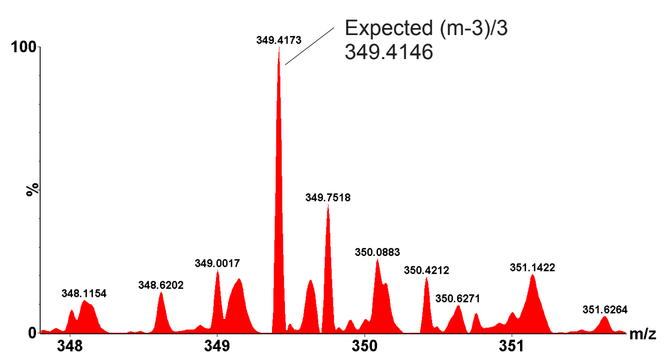

UDP-Mur*N*Ac-tripeptide (Ala:Glu:DAP)

KNOPS +Cb_{100} (C7-8 F20)

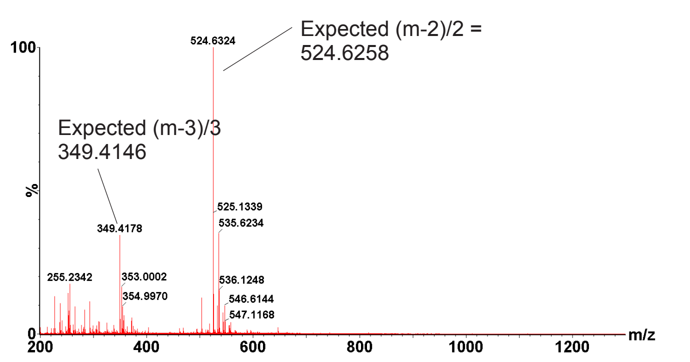

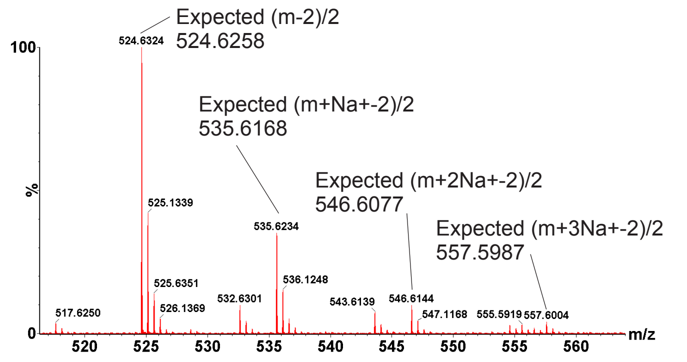

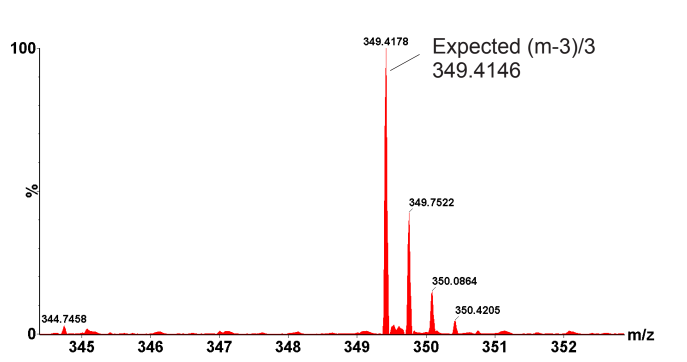

UDP-Mur*N*Ac-pentapeptide (Ala:Glu:DAP:Ala:Ala)

KNOPS alone (C7-C8 F18-19)

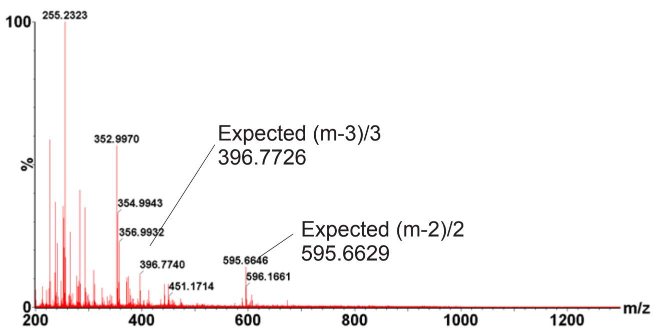

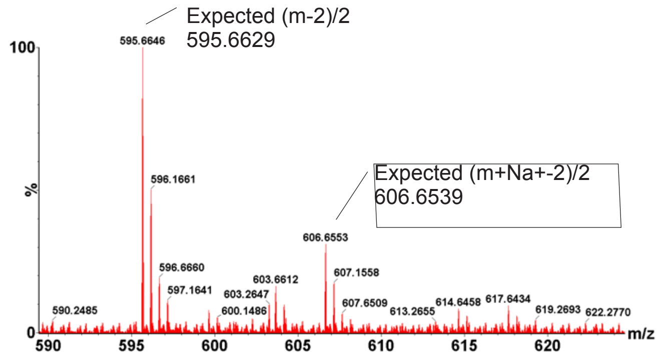

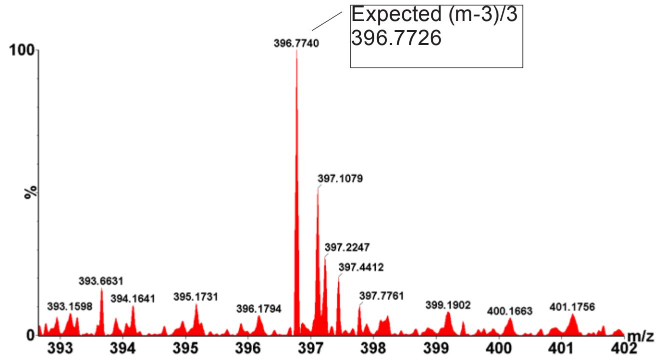

KNOPS +Cb$_{100}$ (C7-8 F19)

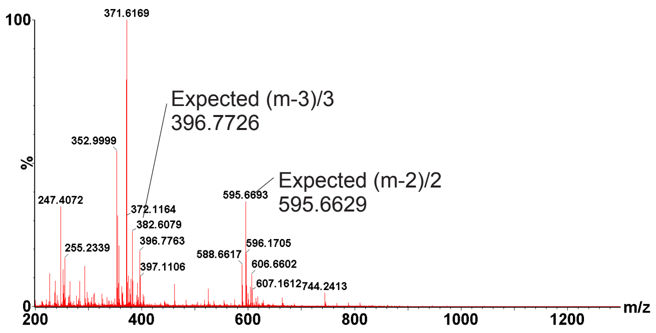

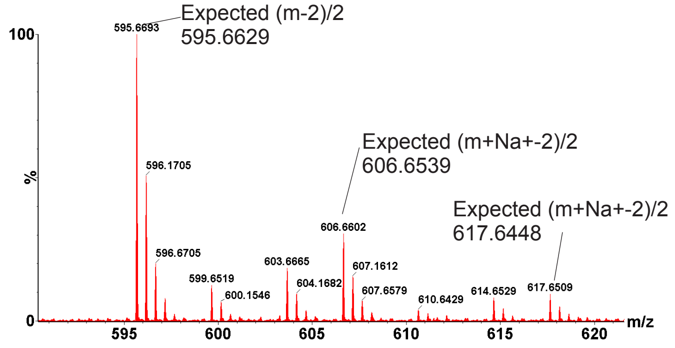

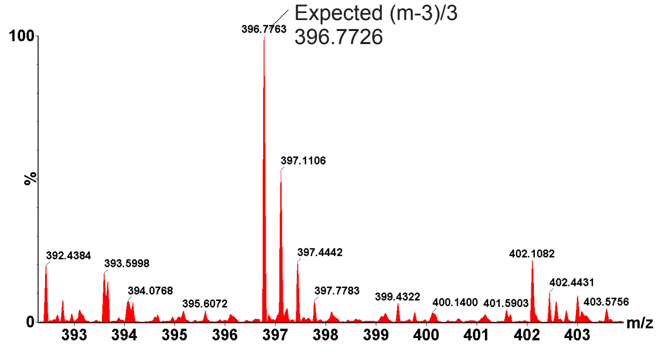

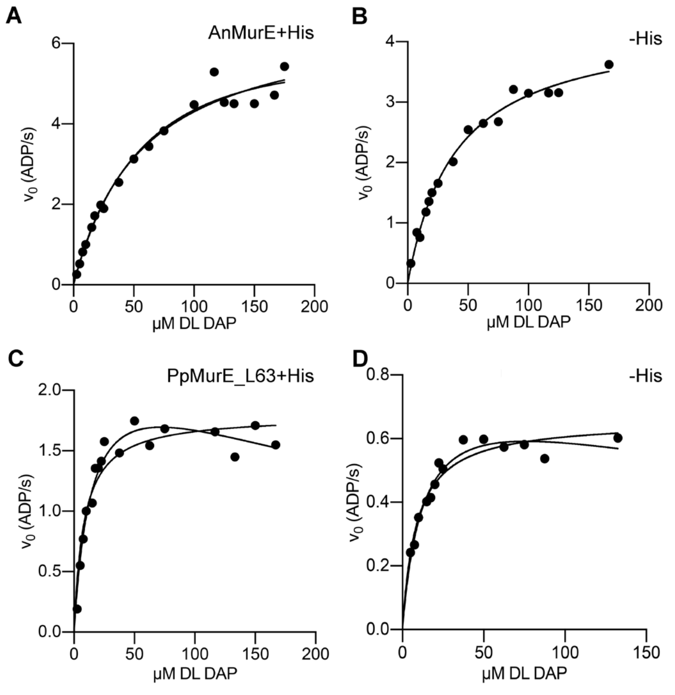

Supplemental Figure S2 D,L-diaminopimelic acid (D,L-DAP) substrate curves for AnMurE and PpMurE_L63. A and C, substrate curves for AnMurE and PpMurE_L63 with His tags, at 50 nM and 146 nM, respectively. B and D, substrate curves for AnMurE and PpMurE_L63 after His tags have been cleaved by TEV protease, at 76 nM and 328 nM, respectively. Assays were with 1 mM UDP-Mur*N*Ac-dipeptide in 50 mM PIPES pH 6.7 (AnMurE) or 50 mM Tricine pH 8.7 (PpMurE_L63). Rates (v_0) in ADP.s^{-1} are mols ADP.mol Mur ligase^{-1}.s^{-1}. Data show Michaelis Menten curves superimposed on those for substrate inhibition and indicate best fit to Michaelis Menten kinetics for AnMurE and to substrate inhibition for PpMurE_L63 (R^2 values for Michaelis Menten and substrate inhibition are in Figure 4, B).

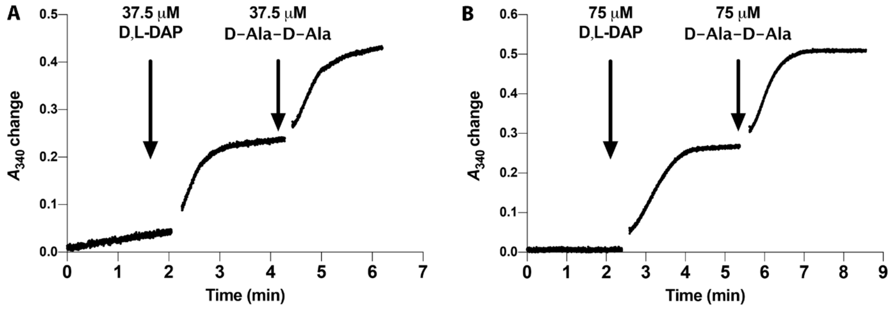

Supplemental Figure S3 Assay data demonstrating *Pseudomonas aeruginosa* MurF (PaMurF) utilizes the products of AnMurE and PpMurE_L63. A, AnMurE and B, PpMurE_L63. Change in NADH absorbance at A_{340} is coupled to the release of ADP by the MurE and MurF ligases on addition of their substrates D,L-DAP and D-Ala--D-Ala, respectively. Assays included 492 nM PaMurF in 50 mM Hepes, pH 7.6, 375 µM UDP-MurNAc-dipeptide and A, 100 AnMurE+His or B, 300 nM PpMurE_L63+His.

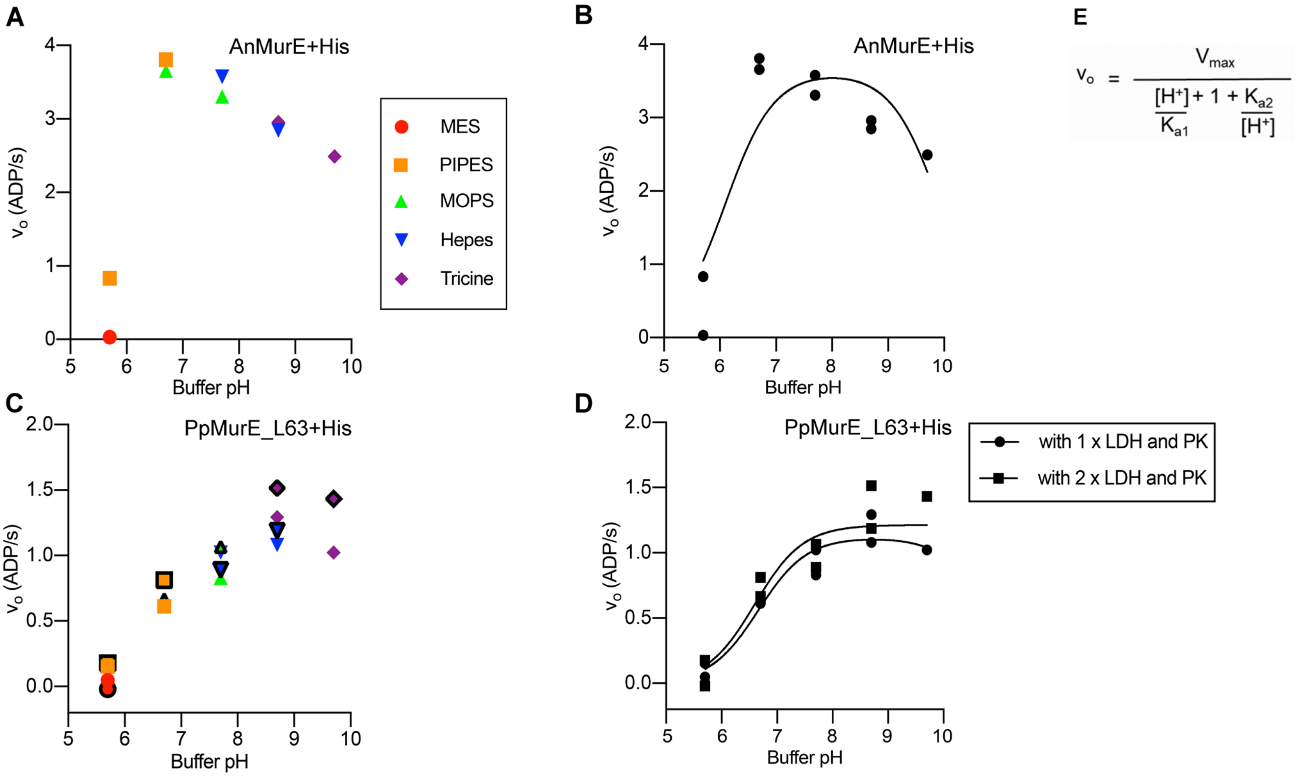

Supplemental Figure S4 Activities of AnMurE and PpMurE_L63 with pH and buffer. Data are presented in two ways: A and C, with coloured symbols to indicate buffers (see legend) and B and D, with non-linear fit to estimate pH optima. Assays for A and B, AnMurE+His and C and D, PpMurE_L63+His, respectively, were in 50 mM buffers in the pH range 5.7-9.7. For PpMurE_L63 C, symbols with black outlines and D, square symbols represent assays with the coupling enzymes lactate dehydrogenase (LDH) and pyruvate kinase (PK) at double the normal concentration (see materials and method), to confirm these were not limiting. Assays included 260 µM UDP-Mur*N*Ac-dipeptide and 150 µM D,L-DAP. If we make the assumption that the only variable responsible for a change in enzyme activity over the pH range tested is the change in [H+] we can derive an equation that follows the relationship of activity *versus* pH (E) where Ka_1 and Ka_2 are dissociation constants of ionizable groups responsible for the ascending and descending limbs of the pH profile. Data indicate that the pH optima for AnMurE and PpMurE_L63 are 7.5 and 7.5-8.5 respectively.

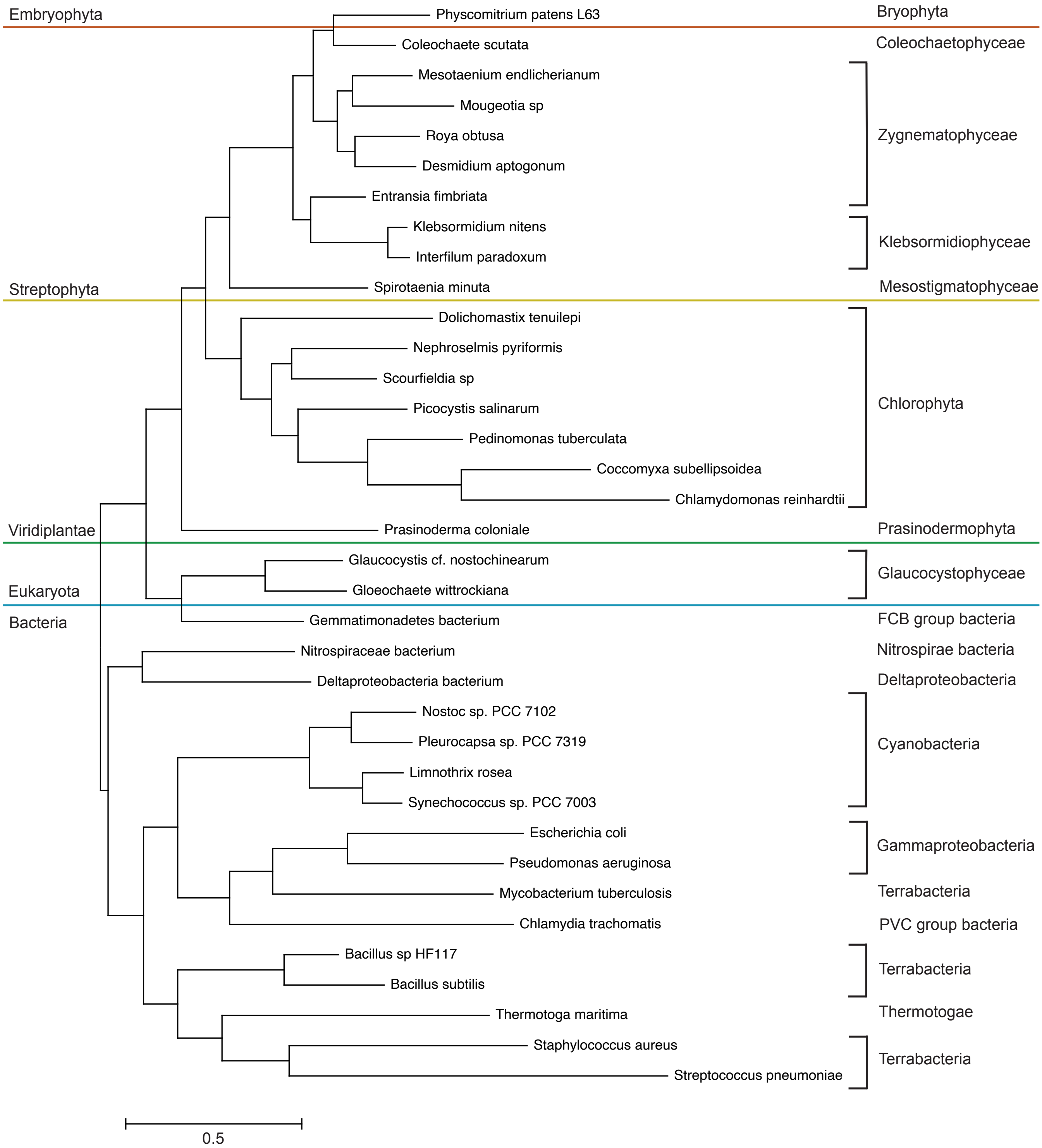

Supplemental Figure S5 Maximum Likelihood phylogram of MurE ancestry using bacterial and algal species with *P. patens* mature MurE protein (PpMurE_L63), computed using ClustalW and MEGA11 software (Tamura et al., 2021). The tree is drawn to scale, with branch lengths measured in the number of substitutions per site. This analysis involved 36 amino acid sequences. There were a total of 1095 positions in the final dataset. Sequences are from Phytozome v13: *P. patens*, Pp3c23_15810V3.2; OneKP: *C. scutata*, VQBJ_2009524; *M. endlicherianum*, WDCW_2047959; *Mougeotia*_sp, ZRMT_2001419; *D. aptogonum*, DFDS_2039413; *R,obtusa*, XRTZ_2003762; *E. fimbriata*, BFIK_2003455; *I. paradoxum*, FPCO_2003655; *S. minuta*, NNHQ_2008964; *D. tenuilepi*, XOAL_2043521; *N. pyriformis*, ISIM_2036943; *Scourfieldia*_sp, EGNB_2030110; *P. salinarum*, TGNL_2009663; *P. tuberculata*, PUAN_2002512; *P. coloniale*, HYHN_2001996; *G. wittrockiana*, PQED_2005637; *G. nostochinearum*, POOW_2010441; NCBI: *K. nitens*, GAQ90408.1; *C. subellipsoidea*, C-16936872; *C. reinhardtii*, Cre12.g519900.t1.2; *Gemmatimonadetes bacterium*, RMH74196.1; *Nitrospiraceae bacterium*, HAK59718.1; *Deltaproteobacteria bacterium*, MBI4208698.1; *Synechococcus* sp. PCC7003, ANV83023.1; *L. rosea,* WP_075891951.1; *Pleurocapsa* sp. PCC7319, WP_019507804.1; *Nostoc* sp. PCC 7102, BAB78029.1; *C. trachomatis*, WP_009872516.1; *M. tuberculosis*, WP_003411189.1; *P. aeruginosa*, WP_043086639.1; *E. coli*, WP_000775093.1; *B. subtilis*, WP_158320450.1; *Bacillus sp. HF117*, WP_160705302.1; *S.aureus*, WP_000340119.1; S. pneumoniae, WP_000590315.1 and Uniprot; *T. maritima*, Q9WY79|. For *C. scutata* and *K. nitens* the estimated mature protein sequences were used, as indicated by alignment to PpMurE_L63. The sequences derived from the OneKP database did not have amino terminal extensions beyond the mature protein.

Jones DT, Taylor WR, Thornton JM (1992) The rapid generation of mutation data matrices from protein sequences. Comput Appl Biosci 8: 275-282
Tamura K, Stecher G, Kumar S (2021) MEGA11: Molecular Evolutionary Genetics Analysis Version 11. Mol Biol Evol 38: 3022-2027

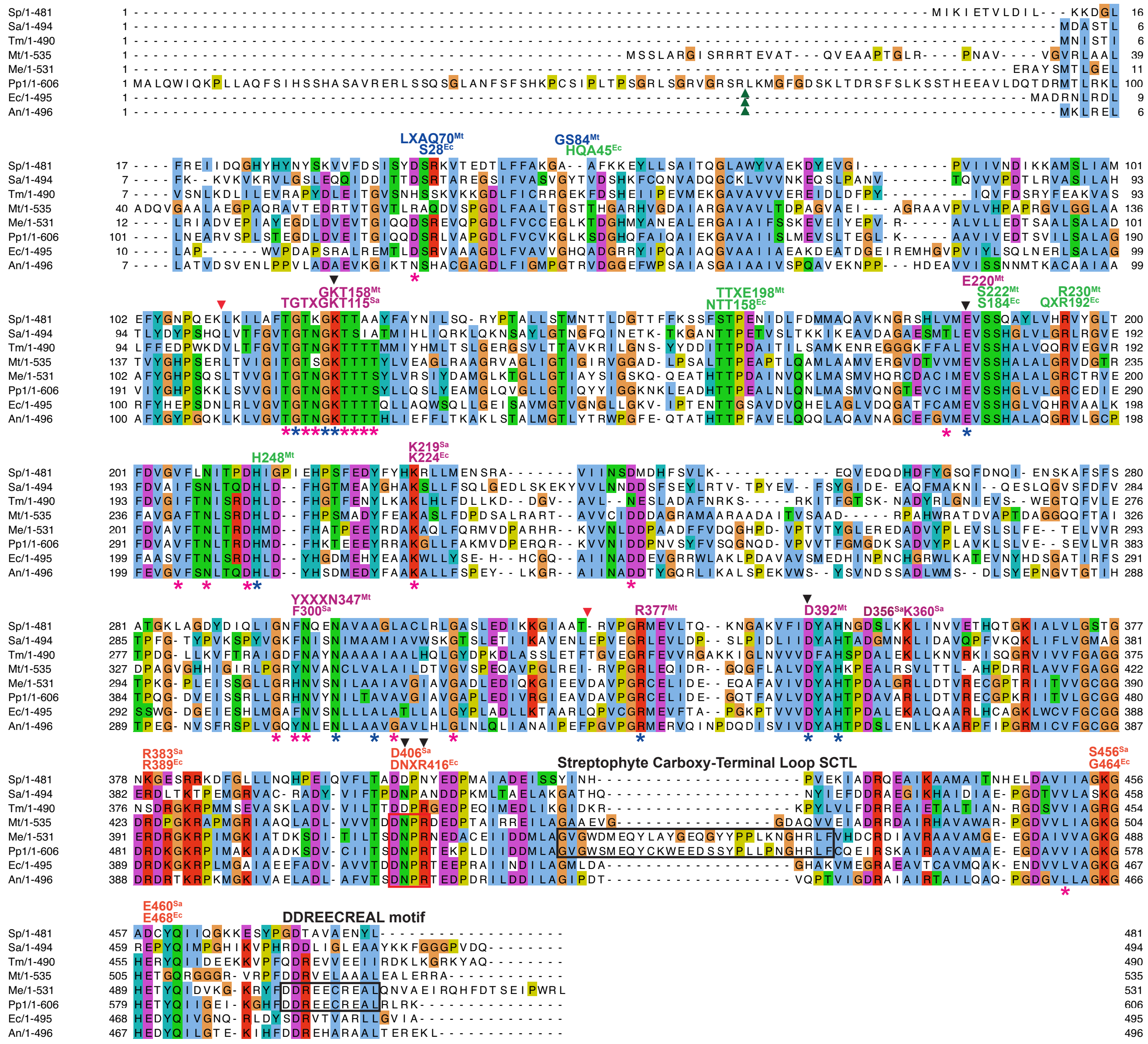

Supplemental Figure S6 Clustal Omega (EMBL-EBI) multiple sequence alignment of MurE homologs displayed using Jalview with ClustalX designated colors (Waterhouse et al., 2009, Madeira et al., 2019): Sp *Streptococcus pneumoniae*, Sa *Staphylococcus aureus*, Mt *Mycobacterium tuberculosum*, Pp1 *P. patens* (Pp3c24_18820V3.2 v3.3 from Phytozome), Tm *Thermotoga maritima,* Me *Mesotaenium endlicherianum* (WDCW from Onekp CNGBDB), Ec *E. coli* (strain K12), An *Anabaena nostoc* PCC7120. All sequences are from the Uniprot or NCBI databases unless stated otherwise. Green arrows indicate ChloroP predicted cleavage site for PpMurE and red arrows the domain hinge points (Smith, 2010). Black arrows indicate residues with a reported role in MtMurE catalysis (Basavannacharya et al 2010). Letter labels indicate numbered residues with published ligand interractions: ^{Ec} for EcMurE (Gordon et al., 2001), ^{Mt} for MtMurE (Basavannacharya et al., 2010; Maitra et al., 2019) and ^{Sa} for SaMurE (Ruane et al., 2013) with colours indicating binding to UDP (blue), Mur*NAc* sugar (green), ATP or ADP (mauve) and DL-DAP (orange) ligands. Blue asterisks indicate residues common to the Mur ligase family, which includes folylpolyglutamate synthetase, cyanophycin synthetase and the capB enzyme from Bacillales (Gordon et al., 2001; Smith, 2010) and pink asterisks indicate residues common to MurC, D, E and F ligases (Basavvanacharya et al., 2010). Two streptophyte-specific features are identified by black boxes and the DNPR consensus by a red box.

Basavannacharya C, Moody PR, Munshi T, Cronin N, Keep NH, Bhakta S (2010) Essential residues for the enzyme activity of ATP-dependent MurE ligase from Mycobacterium tuberculosis. Protein Cell 1: 1011-1022

Gordon E, Flouret B, Chantalat L, van Heijenoort J, Mengin-Lecreulx D, Dideberg O (2001) Crystal structure of UDP-N-acetylmuramoyl-L-alanyl-D-glutamate: meso-diaminopimelate ligase from Escherichia coli. J Biol Chem 276: 10999-11006

Madeira F, Park YM, Lee J, Buso N, Gur T, Madhusoodanan N, Basutkar P, Tivey ARN, Potter SC, Finn RD, Lopez R (2019) The EMBL-EBI search and sequence analysis tools APIs in 2019. Nucleic Acids Res 47: W636-W641

Maitra A, Munshi T, Healy J, Martin LT, Vollmer W, Keep NH, Bhakta S (2019) Cell wall peptidoglycan in Mycobacterium tuberculosis: An Achilles' heel for the TB-causing pathogen. FEMS Microbiol Rev 43: 548-575

Ruane KM, Lloyd AJ, Fulop V, Dowson CG, Barreteau H, Boniface A, Dementin S, Blanot D, Mengin-Lecreulx D, Gobec S, Dessen A, Roper DI (2013) Specificity determinants for lysine incorporation in Staphylococcus aureus peptidoglycan as revealed by the structure of a MurE enzyme ternary complex. J Biol Chem 288: 33439-33448

Smith CA (2006) Structure, function and dynamics in the mur family of bacterial cell wall ligases. J Mol Biol 362: 640-655

Waterhouse AM, Procter JB, Martin DM, Clamp M, Barton GJ (2009) Jalview Version 2--a multiple sequence alignment editor and analysis workbench. Bioinformatics 25: 1189-1191

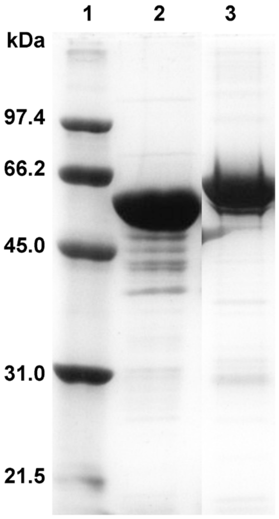

Supplemental Figure S7 PAGE gel of AnMurE and PpMurE_L63 after gel filtration. Lane 1, protein size marker, 2, AnMurE (predicted mass 56.57 kDa) and 3, PpMurE_L63 (predicted mass 62.59 kDa).

Enzyme	Source	K_{cat} (s^{-1})	K_M (µM D,L-DAP/L-Lys)	K_{cat}/K_M ($s^{-1}.mM^{-1}$)
AnMurE+His$^{\text{D,L-DAP}}$	This paper	6.75	56.9	119
AnMurE-His$^{\text{D,L-DAP}}$		4.34	39.4	110
PpMurE_L63+His$^{\text{D,L-DAP}}$		2.41	15.2	159
PpMurE_L63-His$^{\text{D,L-DAP}}$		0.793	13.0	61.0
PaMurE$^{\text{D,L-DAP}}$	(Paradis-Bleau et al., 2009)	22.2	140	160*
CtMurE$^{\text{D,L-DAP}}$	(Patin et al., 2009)	0.233	23.0	10.1
MtMurE$^{\text{D,L-DAP}}$	(Munshi et al., 2013)	1.2	69.0	17.4
EcMurE$^{\text{D,L-DAP}}$	(Patin et al., 2010)	1.24*	40.0	31.1
SaMurE$^{\text{L-Lys}}$	(Patin et al., 2010)	4.79*	550	8.71
SaMurE$^{\text{L-Lys}}$	(Ruane et al., 2013)	4.83	550	8.79
TmMurE$^{\text{L,Lys}}$	(Boniface et al., 2006)	24.6*	2800	10.3
TmMurE$^{\text{D,L-DAP}}$		4.38*	4800	0.912

Supplemental Table S1 Comparison of AnMurE and PpMurE_L63 kinetics with D,L-diaminopimelic acid (D,L-DAP) with published data for other MurE ligases. PaMurE$^{\text{D,L-DAP}}$ *Pseudomonas aeruginosa*, CtMurE$^{\text{D,L-DAP}}$ *Chlamydia trachomatis*, MtMurE$^{\text{D,L-DAP}}$ *Mycobacterium tuberculosis*, EcMurE$^{\text{D,L-DAP}}$ *Escherichia coli*, SaMurE$^{\text{L-Lys}}$ *Staphylococcus aureus* and TmMurE$^{\text{L-Lys}}$ *Thermotoga maritima*. Asterisks indicate where data were extrapolated from the published values.

Boniface A, Bouhss A, Mengin-Lecreulx D, Blanot D (2006) The MurE synthetase from Thermotoga maritima is endowed with an unusual D-lysine adding activity. J Biol Chem 281: 15680-15686

Munshi T, Gupta A, Evangelopoulos D, Guzman JD, Gibbons S, Keep NH, Bhakta S (2013) Characterisation of ATP-dependent Mur ligases involved in the biogenesis of cell wall peptidoglycan in Mycobacterium tuberculosis. PLoS One 8: e60143

Paradis-Bleau C, Lloyd A, Sanschagrin F, Maaroufi H, Clarke T, Blewett A, Dowson C, Roper DI, Bugg TD, Levesque RC (2009) Pseudomonas aeruginosa MurE amide ligase: enzyme kinetics and peptide inhibitor. Biochem J 421: 263-272

Patin D, Boniface A, Kovac A, Herve M, Dementin S, Barreteau H, Mengin-Lecreulx D, Blanot D (2010) Purification and biochemical characterization of Mur ligases from Staphylococcus aureus. Biochimie 92: 1793-1800

Patin D, Bostock J, Blanot D, Mengin-Lecreulx D, Chopra I (2009) Functional and biochemical analysis of the Chlamydia trachomatis ligase MurE. J Bacteriol 191: 7430-7435

Ruane KM, Lloyd AJ, Fulop V, Dowson CG, Barreteau H, Boniface A, Dementin S, Blanot D, Mengin-Lecreulx D, Gobec S, Dessen A, Roper DI (2013) Specificity determinants for lysine incorporation in Staphylococcus aureus peptidoglycan as revealed by the structure of a MurE enzyme ternary complex. J Biol Chem 288: 33439-33448
